# *In situ* X‑ray Synchrotron Studies Reveal the Nucleation and Topotactic Transformation of Iron Sulfide Nanosheets

**DOI:** 10.1021/jacs.5c15843

**Published:** 2025-12-12

**Authors:** Cecilia A. Zito, Lars Klemeyer, Francesco Caddeo, Brian Jessen, Sani Y. Harouna-Mayer, Lise-Marie Lacroix, Malte Langfeldt, Tjark L. R. Gröne, Jagadesh K. Kesavan, Chia-Shuo Hsu, Alexander Schwarz, Ann-Christin Dippel, Fernando Igoa Saldaña, Blanka Detlefs, Dorota Koziej

**Affiliations:** † 98889 University of Hamburg, Institute for Nanostructure and Solid-State Physics, Center for Hybrid Nanostructures, Luruper Chaussee 149, Hamburg 22761, Germany; ‡ The Hamburg Center for Ultrafast Imaging, Hamburg 22761, Germany; § Laboratoire de Physique et Chimie des Nano-Objets, 137668 UMR 5215 INSA, CNRS, UPS, Université de Toulouse, 135 avenue de Rangueil, Toulouse Cedex 4 F-31077, France; ∥ Institut Universitaire de France (IUF), 103 Boulevard Saint Michel, Paris 75005, France; ⊥ 14915 University of Hamburg, Institute for Nanostructure and Solid-State Physics, Jungiusstraße 11a, Hamburg 20355, Germany; # Deutsches Elektronen-Synchrotron DESY, Notkestraße 85, Hamburg 22607, Germany; ∇ ESRF, The European Synchrotron Facility, 71 Avenue des Martyrs, CS40220, Grenoble Cedex 9 38043, France

## Abstract

Iron sulfides (Fe_*x*_S_*y*_), including greigite (Fe_3_S_4_), are key materials in geological processes and technological applications. However, in the context of colloidal synthesis, the mechanism by which these nanoparticles form remains unexplored. Here, we employ *in situ* X-ray diffraction and photon-in photon-out spectroscopic studies to elucidate the reaction pathway of Fe­(acac)_3_ and thioacetamide (TAA) in benzyl alcohol (BA), which yields crumpled Fe_3_S_4_ nanosheets. Using powder X-ray diffraction (PXRD), we identify FeS (mackinawite) as a crystalline intermediate whose anisotropic growth, driven by its layered crystal structure, governs the crumpled nanosheet-like morphology of Fe_3_S_4_ (greigite) through a topotactic transition. By performing high-resolution fluorescence-detected X-ray absorption near-edge structure (HERFD-XANES) spectroscopy, we show that the formation of Fe_3_S_4_ proceeds through a multistep mechanism involving two intermediates. Supported by density functional theory (DFT), we find that Fe­(acac)_3_ is initially reduced in the presence of TAA in BA, forming a molecular intermediate [Fe­(acac)_2_(BA)_2_], which subsequently transforms into FeS and ultimately into Fe_3_S_4_. Complementary valence-to-core X-ray emission spectroscopy (vtc-XES) reveals the evolution of the coordination environment from Fe–O to Fe–S throughout the reaction. Our work provides a comprehensive understanding of the formation mechanism of Fe_3_S_4_ nanosheets in solution, shedding light on how crystal growth dynamics and electronic structure evolution dictate their unique crumpled nanosheet morphology.

## Graphical abstract

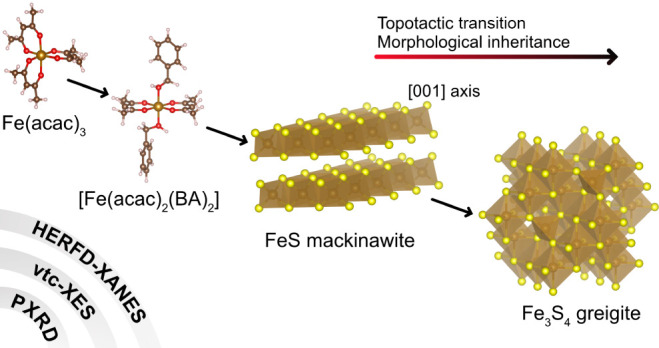

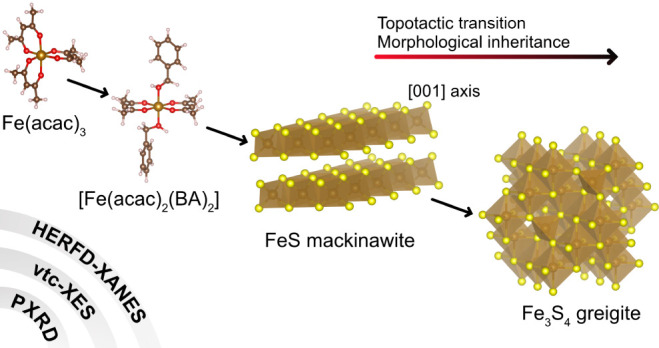

## Introduction

Iron sulfides (Fe_*x*_S_*y*_) have attracted attention because of their importance in geological and environmental processes and also for their technological applications. Numerous Fe_*x*_S_*y*_ phases occur naturally as minerals, including FeS_2_ (pyrite), m-FeS_2_ (marcasite), FeS (troilite), FeS (mackinawite), Fe_3_S_4_ (greigite), Fe_3+*x*_S_4_ (smythite), and Fe_1–*x*_S (pyrrhotite),^1^ which are abundant and play an important role in the sulfur cycle and in understanding Earth’s surface transformations.^1–3^ Some metastable phases undergo chemical transformation to form more stable ones. For instance, geoscience studies have revealed the formation of pyrite via mackinawite and greigite as intermediates in anoxic conditions.^4,5^ Fe_*x*_S_*y*_ compounds are also environmentally friendly base materials for energy storage,^6–8^ electrocatalysis,^9,10^ photoelectrochemistry,^11^ photovoltaics,^12^ and magnetic applications.^13,14^ However, their full potential applications remain to be explored. Theoretical calculations predict a Verwey transition for greigite, the sulfur analog of magnetite, which has not yet been observed experimentally.^15,16^

The focus in colloidal synthesis has been on identifying synthetic parameters yielding a specific Fe_*x*_S_*y*_ phase.^17–19^ In particular, the synthesis of greigite nanostructures is typically carried out via solvothermal^20^ or heat-up methods under inert atmosphere conditions,^8,18,21^ usually in nonpolar solvents. Here, we developed a simple and fast solvothermal reaction, without the need for an anoxic or inert atmosphere, to synthesize Fe_3_S_4_ (greigite) nanosheets. The method involves reacting iron­(III) acetylacetonate (Fe­(acac)_3_) with thioacetamide (TAA) as a sulfur source in benzyl alcohol (BA) at 180 °C. Additionally, the use of a polar organic solvent opens up the possibility of conducting microwave-assisted reactions.^22^

We combine *in situ* powder X-ray diffraction (PXRD), high-resolution fluorescence detected X-ray absorption near-edge structure (HERFD-XANES), and valence-to-core X-ray emission spectroscopy (vtc-XES) to elucidate the reaction mechanism of crumpled Fe_3_S_4_ nanosheets. PXRD enables the characterization of nanoparticle crystallization and growth, anisotropy, phase transitions, and the identification of crystalline intermediates.^23–28^ HERFD-XANES is an element-selective tool for monitoring changes in the coordination environment and oxidation state of the absorbing metal center,^29,30^ and has been employed to identify the formation of metal complexes, including those arising from interaction with capping agents, and noncrystalline nuclei in solution.^31–34^ Complementarily, vtc-XES probes the nature of ligands coordinated to the absorbing atom,^35–37^ and has emerged as a powerful characterization technique for studying metal complexes and catalysts.^38–40^ As shown in Figure 1, we reveal that the unique nanosheet-like morphology of Fe_3_S_4_ (greigite) is dictated by a topotactic transition, in which the preferential basal plane growth of the metastable layered FeS (mackinawite) intermediate determines the nanosheet morphology that is subsequently transferred to the emerging cubic spinel phase. Prior to the FeS formation, we identify the reduction of the Fe­(acac)_3_ precursor accompanied by the coordination of two solvent molecules, yielding the molecular intermediate [Fe­(acac)_2_(BA)_2_]. Analysis of the pre-edge region of the HERFD-XANES spectra provides insights into the coordination chemistry of these molecular complexes, including a direct experimental determination of the crystal-field splitting parameter.^41,42^ Therefore, we monitor the full evolution of the coordination environment from Fe–O to Fe–S: Fe­(acac)_3_ is reduced to [Fe­(acac)_2_(BA)_2_], which then converts into FeS and ultimately to Fe_3_S_4_.

**Figure 1:**
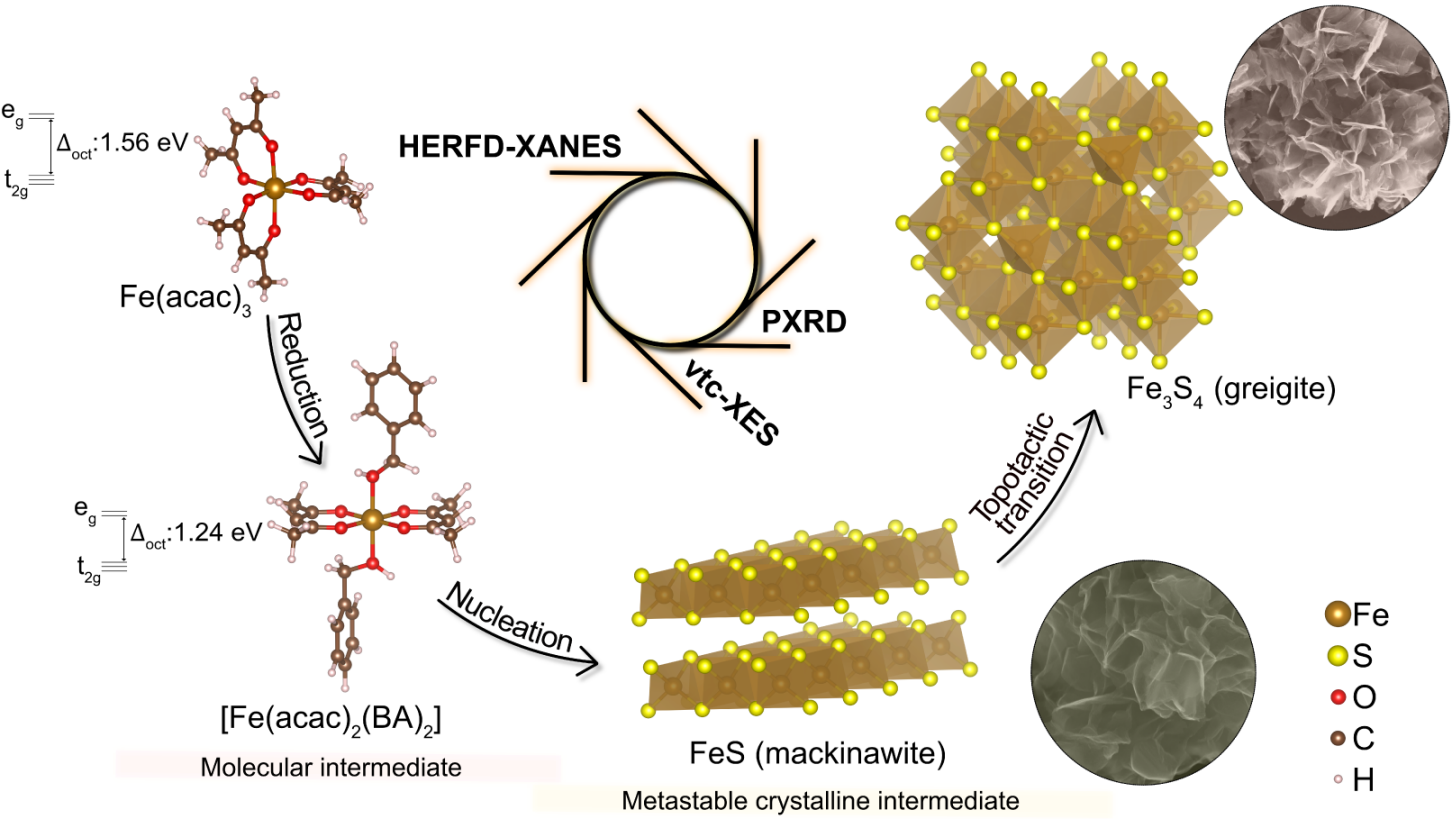
Schematic illustration of the *in situ* X-ray studies for monitoring the formation of Fe_3_S_4_ nanosheets in solution. HERFD-XANES, vtc-XES, and PXRD provide complementary information to track the processes taking place during the colloidal reaction, providing the full picture of the chemical reaction pathway. Δ_oct_ (also known as 10Dq) refers to the crystal-field splitting parameter in an octahedral field. BA stands for benzyl alcohol.

## Results and Discussion

We synthesize Fe_3_S_4_ nanosheets via a solvothermal reaction between Fe­(acac)_3_ and TAA in BA at 180 °C without the need of inert or anoxic conditions. The scanning electron microscopy (SEM) images in Figure 2a,b reveal the formation of thin, nanosheet-like structures that exhibit notable wrinkling or crumpling and are interconnected, assembling into a flower-like morphology. Moreover, the surface of the nanosheets is rough, with sharp edges and folds observed. In Figure 2c, the transmission electron microscopy (TEM) image further confirms the two-dimensional character of the nanostructures. A larger area imaged in the TEM image of Figure S1a reveals the presence of smaller particles alongside the nanosheet-like structures. The increased transparency in certain regions highlights the thin structure of the nanosheets. The individual nanosheets exhibit a thickness ranging from 14 to 38 nm. Figure 2d presents the high-resolution TEM (HRTEM) image of a Fe_3_S_4_ nanosheet along with the corresponding fast Fourier transform (FFT) pattern, which shows the (311) and (533) planes from Fe_3_S_4_ (greigite). These planes are further illustrated in Figure 2e,f, where the marked regions from Figure 2d are magnified to highlight the lattice fringes associated with each plane. The selected area electron diffraction (SAED) pattern, corresponding to the area imaged in Figure S1b, reveals the polycrystalline nature of the sample, with diffraction rings corresponding to the planes of the cubic spinel structure of Fe_3_S_4_ (Figure S1c). In Figure 2g–i, the energy-dispersive X-ray spectroscopy (EDX) mapping shows the even distribution of Fe and S along the nanoparticles. Semi-quantitative EDX analysis yields an approximate S:Fe atomic ratio of 1.35, which is close to the empirical value of 1.33 (Figure S1d). As shown in Figures 2j and S2, the atomic force microscopy (AFM) image of a single Fe_3_S_4_ nanosheet exhibits a thickness of approximately 14 nm, which is in good agreement with the TEM measurements. Complementary characterization of the thermal stability and magnetic properties of Fe_3_S_4_ nanosheets is displayed in Figures S3,4, Supplementary Notes 1 and Table S1.^43–48^

**Figure 2:**
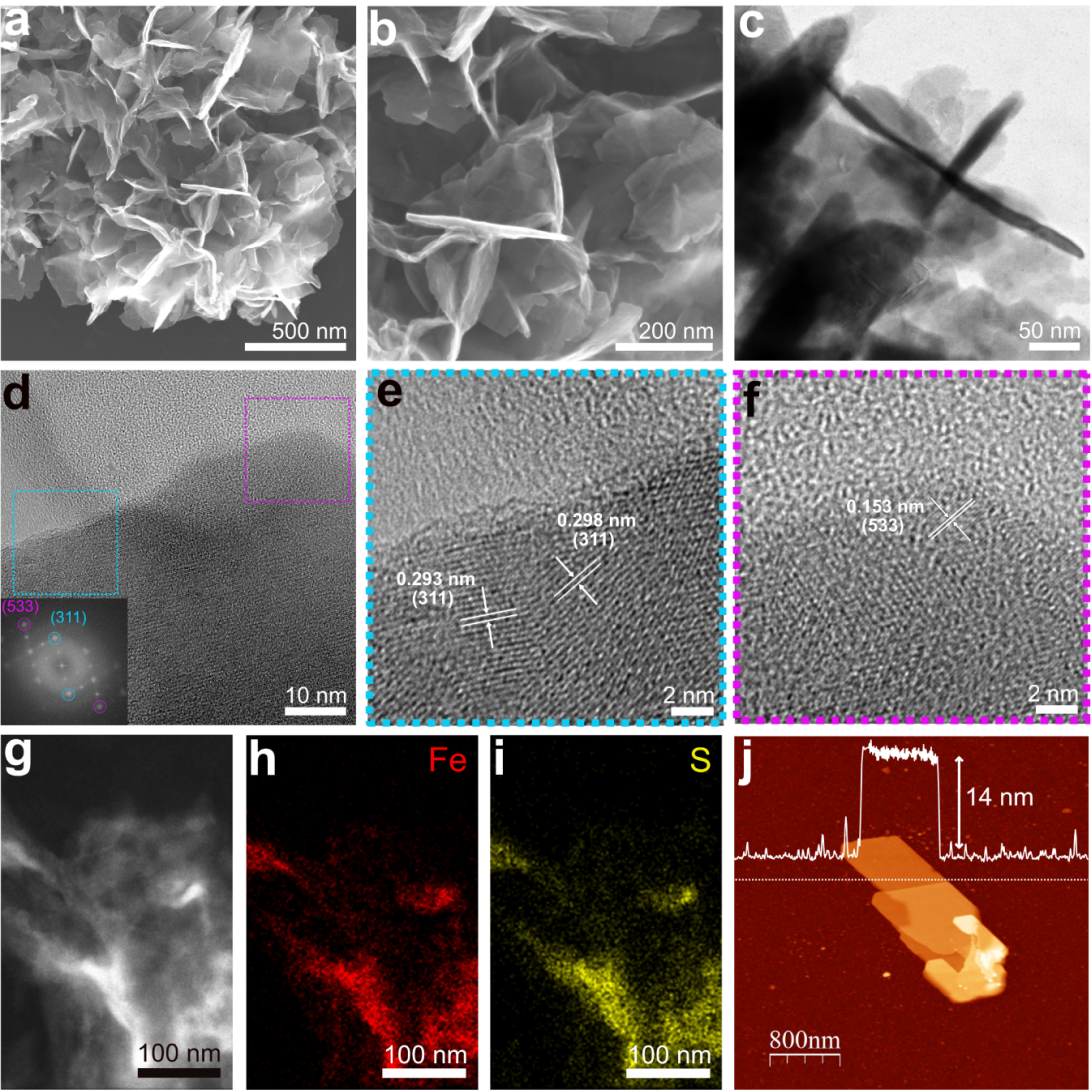
Morphological characterization of the Fe_3_S_4_ nanosheet-like structures. (a,b) SEM images at different magnifications. (c) TEM image, evidencing the two-dimensional structure, (d) HRTEM image with the corresponding FFT pattern shown in the inset. (e,f) Magnified views of the boxed regions marked in blue and pink in (d), highlighting the crystallographic planes of Fe_3_S_4_. (g−i) Dark-field STEM image and the corresponding EDX elemental mapping of Fe (red) and S (yellow). (j) AFM image and corresponding height profile of a Fe_3_S_4_ nanosheet on a Si wafer. The dashed line indicates the region where the profile was measured.

The predominant nanosheet-like morphology of the Fe_3_S_4_ particles, despite their cubic inverse spinel structure, suggests a complex formation mechanism, involving anisotropic growth or morphological inheritance from a layered-structured precursor. To unravel the reaction pathway leading to the formation of crumpled Fe_3_S_4_ nanosheets, we employ *in situ* X-ray diffraction and spectroscopic studies.

### *In Situ* PXRD Uncovers Topotactic Transformation of FeS to Fe_3_S_4_ that Governs Nanosheet Morphology

We perform *in situ* PXRD studies during the synthesis of Fe_3_S_4_ nanosheets at 180 °C to elucidate their crystallization in solution. Figure 3a shows the time-resolved, background-subtracted *in situ* PXRD data. At the early stages of heating, only a peak at *q* values below 2 Å^–1^ related to residual background is observed, excluding the existence of crystalline species in the reaction. The first reflection, appearing at *q* ∼ 3.4 Å^–1^ when the temperature reaches about 120 °C, corresponds to the (200) reflection of tetragonal FeS (mackinawite). Subsequently, additional reflections emerge at *q* ∼ 2.4, 2.7, and 4.8 Å^–1^, which can be assigned to the (110), (111) and (220) planes of FeS, respectively. The sharpness and high intensity of the (200) reflection, compared to the others, indicate anisotropy in the FeS nanoparticle’s shape, as confirmed by the Rietveld refinement discussed below. Upon reaching the final reaction temperature of 180 °C, the characteristic peaks of Fe_3_S_4_ (greigite) appear. The intensity of Fe_3_S_4_ peaks increases concomitantly with the decrease in intensity of the reflections of FeS, indicating the transformation of FeS into Fe_3_S_4_. As the reaction further progresses, Fe_3_S_4_ becomes the predominant crystalline phase, persisting until the end of the reaction after 75 min (≡ 60 min at 180 °C).

**Figure 3:**
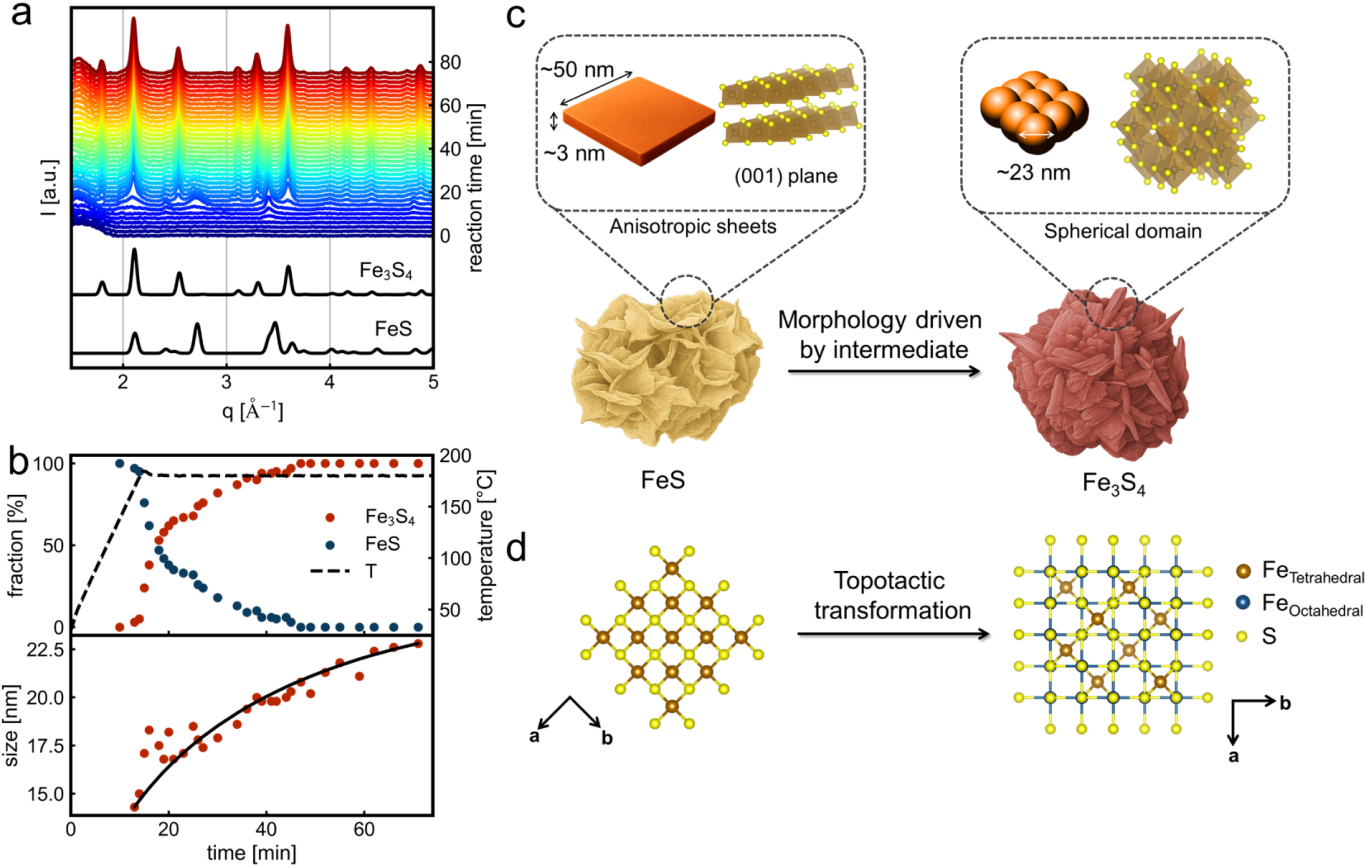
Crystalline phases formation. (a) *In* situ time-resolved PXRD patterns for the synthesis of Fe_3_S_4_ nanosheets at 180 °C at 10 °C/min, compared with the standard patterns for greigite Fe_3_S_4_ (*Fd3̅m*) and mackinawite FeS (*P4/nmm*) phases. (b) Results from the sequential Rietveld refinement. Top: evolution of the weight fraction of FeS (mackinawite) and Fe_3_S_4_ (greigite) over time. Bottom: crystallite size evolution of Fe_3_S_4_ (spherical model). The solid line shows an empirical growth trend. (c) Schematic illustration of the preferential growth of FeS within the (001) plane, yielding the nanosheet-like morphology, which is mostly preserved during the transformation to Fe_3_S_4_ that presents spherical domain size. (d) Illustration of the topotactic transformation of FeS to Fe_3_S_4_, whereby the transformation in solid state allows for preserving the cubic close-packed-like substructure within the crystals. Structures from the crystallographic data of Fe_3_S_4_ (ICSD-42535) and FeS (ICSD-81087) were illustrated using Vesta software.^50^

We performed sequential Rietveld refinement on the *in situ* PXRD to monitor the evolution and contributions of the two Fe_*x*_S_*y*_ phases, along with crystal size and isotropy, throughout the reaction. As shown in the top part of Figure 3b, the refinement confirms the gradual yet fast decrease in the FeS fraction with the concomitant increase in the Fe_3_S_4_ fraction as the reaction progresses. The FeS contribution converges to zero after 47 min reaction time, and the final product at 75 min (60 min at 180 °C) matches well with only a single-phase, Fe_3_S_4_, as evidenced by the Rietveld refinement in Figure S5.

Additionally, the domain size of Fe_3_S_4_ increases from approximately 14 nm (178 °C) at the early stages of the reaction to ∼ 22 nm by the end of the reaction at 75 min (bottom of Figure 3b). While the refinement of the PXRD data using a spherical domain model effectively describes the Fe_3_S_4_ contribution, a uniaxial model with [001] as the unique axis is required for the FeS phase. Consequently, we observe an anisotropic plate-like structure for FeS, with an average thickness (axial size) of 3 nm and equatorial size of ∼ 50 nm during the initial 30 min of reaction (see Table S2). This suggests that FeS (mackinawite) grows preferentially within the (001) plane (i.e., the basal plane), where edge-sharing FeS_4_ tetrahedra can form extended layers, as previously evidenced.^49^ Therefore, the nanosheet-like structure of Fe_3_S_4_ results from the anisotropic growth of the FeS (mackinawite) intermediate, driven by its layered crystal structure, rather than any inherent structural anisotropy in Fe_3_S_4_ (Figure 3c).

To verify this hypothesis, we lower the synthesis temperature to 140 °C, which slows the phase transition of FeS to Fe_3_S_4_ (Figure S6). Even after 180 min, the product remains a mixture of both crystalline phases, with FeS (mackinawite) as the dominant phase. As shown in Figure S7, the morphology at 140 °C corresponds to highly crumpled, interconnected thin nanosheets (15 nm thick), with a significant degree of assembly. These nanosheets likely arise from the preferential growth of FeS along the basal plane. We thus propose that the phase transformation from FeS to Fe_3_S_4_ follows a topotactic mechanism, in which the crystalline solid undergoes structural reorganization due to partial oxidation of Fe^2+^ to Fe^3+^, and rearrangement of sulfur and iron ions, while partially preserving the crystal substructure.^4,50^ Figure 3d depicts the crystal structures of both phases, oriented to highlight their cubic close-packed-like substructure. This process enables the material to retain its initial morphology to a certain extent while transforming from FeS to Fe_3_S_4_, resulting in an intermediate-imposed morphology. Similar findings are seen for the reaction at 160 °C (see Figures S6 and S8).

### Tracking the Electronic Structure Evolution Using HERFD-XANES and vtc-XES

The PXRD reveals the formation of phase-pure cubic inverse spinel Fe_3_S_4_ nanosheets with tetragonal FeS as a crystalline intermediate. However, it fails to provide information at the molecular level before the crystallization of the nanoparticles and on the evolution of the electronic structure throughout the reaction. Hence, we conduct combined *in situ* Fe vtc-XES and Fe K-edge HERFD-XANES measurements during the synthesis of Fe_3_S_4_ to track all the chemical changes surrounding the Fe atoms by probing the occupied and unoccupied states, respectively (Figure 4a). Figure 4b schematically shows the electronic transitions giving rise to the observed features in each technique. To slow down the reaction kinetics and to obtain more detailed information into the reaction dynamics, we record both vtc-XES and HERFD-XANES spectra at a heating rate of 1 °C/min up to 180 °C.

**Figure 4:**
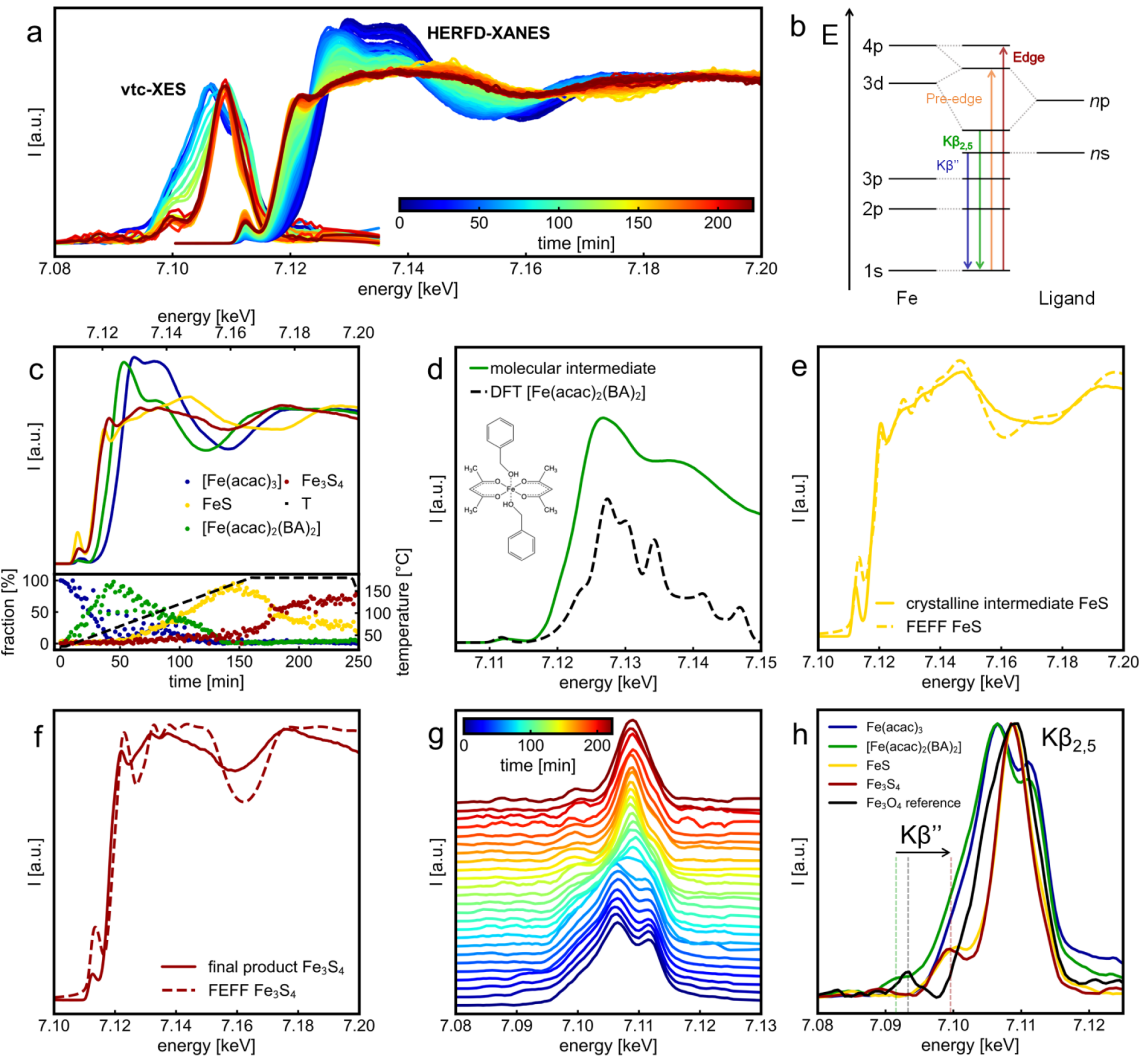
*In situ* spectroscopic studies during the synthesis of Fe_3_S_4_ nanosheets at 180 °C with heating rate of 1 °C/min. (a) Combined *in situ* Fe vtc-XES and Fe K-edge HERFD-XANES measurements. (b) Schematic illustration of the energy diagram with the transitions that originate the vtc-XES and Fe K-edge features. (c) Spectra of the recovered individual components by MCR-ALS analysis and their relative concentrations over reaction time. The black dashed line (T) represents the reaction temperature profile. (d) Comparison of the recovered spectrum of the 2nd compound (molecular intermediate) with the theoretical spectrum of [Fe­(acac)_2_(BA)_2_] complex from DFT calculations. The theoretical spectrum was shifted by 25.0 eV to align with the experimental data. BA corresponds to benzyl alcohol. (e) Recovered spectrum from MCR-ALS analysis for the 3rd compound, assigned to the metastable intermediate FeS (mackinawite), compared with the theoretical XANES spectrum from FEFF calculations for FeS. (f) Comparison of the recovered spectrum of the 4th compound, corresponding to the final product Fe_3_S_4_ (greigite), with the theoretical XANES spectrum calculated using FEFF for Fe_3_S_4_. (g) Waterfall plot of the i*n situ* vtc-XES data set shown in (a). (h) Selected vtc-XES spectra corresponding to the four individual components during the Fe_3_S_4_ synthesis: Fe­(acac)_3_, [Fe­(acac)_2_(BA)_2_] complex, FeS (mackinawite), and Fe_3_S_4_ (greigite), compared to Fe_3_O_4_ (magnetite) as a reference for the crystalline oxide analogue of Fe_3_S_4_.

In the HERFD-XANES spectra, we observe that upon heating the reaction, the onset of the absorption edge shifts to lower energy values, and the white line features show a decrease in intensity, suggesting the reduction of Fe^3+^ in the precursor and a possible change in the coordination of atoms. Similarly, the pre-edge features shift to lower energies and subsequently exhibit an increase in intensity. In particular, the pre-edge reaches its maximum intensity when the reaction temperature reaches 180 °C, then gradually decreases in intensity, accompanied by a shift back to higher energy as the reaction progresses. Since the pre-edge corresponds to the dipole forbidden 1*s*→3*d* transition, high intensities are associated with a higher degree of hybridization between the unoccupied 3*d* and 4*p* orbitals, typically due to deviations from a perfectly centrosymmetric coordination. Accordingly, the low intensity pre-edge seen at the beginning of the reaction is consistent with the octahedral coordination of Fe­(acac)_3_. In contrast, the shift to lower energy and rise in intensity at around 180 °C correspond to the formation of FeS (mackinawite) intermediate, in which Fe^2+^ cations are solely tetrahedrally coordinated. The subsequent decrease in intensity and shift to higher energy indicate the conversion to Fe_3_S_4_, where only one-third of Fe^3+^ ions are in tetrahedral symmetry, while the remaining Fe^2+^/Fe^3+^ ions occupy octahedral sites. A more in-depth analysis of the pre-edge features is discussed later.

**Figure 5:**
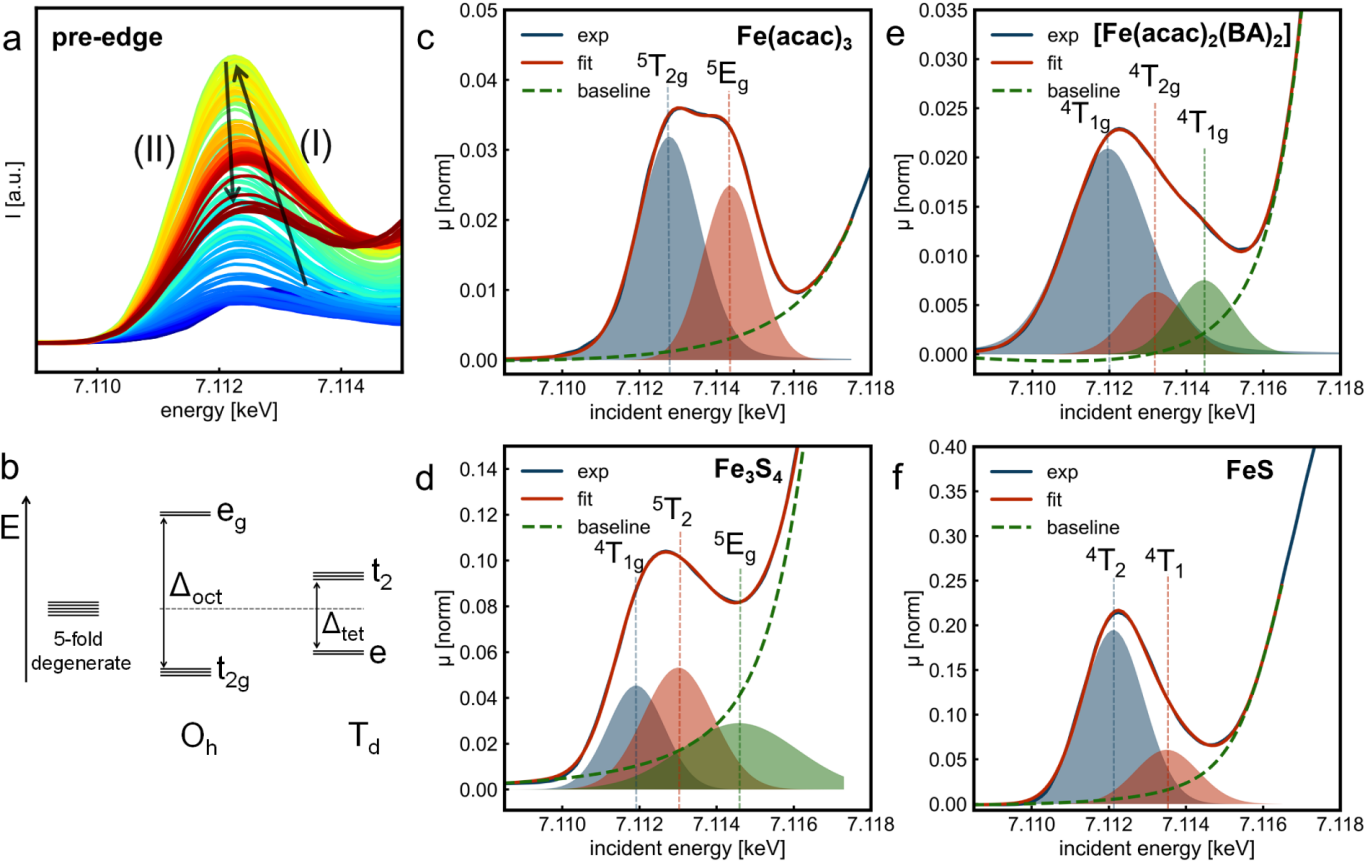
Analysis of the pre-edge features. (a) Full data set of the pre-edge region of the *in situ* HERFD-XANES spectra, where the arrow (I) shows the increase in the feature intensity and (II) indicates the decrease in intensity for the final product. The time scale is the same of Figure 4a. (b) Schematic illustration of the crystal field splitting of *d* orbitals according to the O_h_ and *T*_d_ symmetries. (c–f) Fit to the Fe K-edge pre-edge feature of the individual recovered spectra from MCR-ALS analysis for (c) precursor Fe­(acac)_3_—first recovered compound, (d) final product Fe_3_S_4_—last recovered component, (e) molecular intermediate [Fe­(acac)_2_(BA)_2_] complex—second recovered compound, and (f) FeS mackinawite as crystalline intermediate—third recovered compound. Experimental data are shown in blue, fit in red, and background function shown in dashed green lines. All the peaks were fitted with pseudo-Voigt functions.

To understand the qualitative observations from the HERFD-XANES data, we perform multivariate curve resolution by alternating least-squares (MCR-ALS) analysis, extracting information on the independent components during the *in situ* reaction. Details of the method are given in Supplementary Notes 2, Figure S9, Tables S3 and 4. MCR-ALS analysis allows us to identify four independent components in our reaction, and their recovered spectra along with their relative concentration over time are shown in Figure 4c. The recovered component at the start of the reaction (blue curve) has the maximum of the first derivative of the absorption spectrum (E_0_) at 7127.0 eV and reproduces well the features of the Fe­(acac)_3_ reference (see Figure S10). Thus, no significant changes occur to the Fe­(acac)_3_ precursor when dissolved together with TAA in BA prior to heating.

The second recovered compound (shown in green) displays an E_0_ at 7123.2 eV, corresponding to a 3.8 eV shift to lower energy relative to the first component—Fe­(acac)_3_, indicating a reduction to Fe^2+^. Yet, the edge position is not as low in energy as those observed for the third and fourth recovered compounds, which appear later in the reaction. These latter two components likely correspond to the Fe_*x*_S_*y*_ crystalline phases identified by *in situ* PXRD. We thus hypothesize that the coordination around Fe in the second compound is dominated by oxygen ligands rather than sulfur. To uncover the nature of the first reaction intermediate, we simulate theoretical XANES spectra of plausible intermediate compounds using density functional theory (DFT) calculations in ORCA. Figure 4d shows good agreement between the experimental data (green line) and the calculated XANES spectrum for the molecular complex [Fe­(acac)_2_(BA)_2_], in which the Fe^2+^ ion is coordinated in an octahedral symmetry by oxygen atoms from two acetylacetonate ligands and two solvent molecules. The spectral shape and features of experimental data cannot be reproduced by the other proposed compounds (Figure S11). In a similar reaction in BA, we previously identified an octahedrally coordinated Co­(acac)_2_ with two solvent molecules as an intermediate species.^31^ However, BA does not act as the sole reducing agent, and we hypothesize that the thermal decomposition of TAA also contributes to the reduction process.

The third recovered component (yellow curve) has a shift in E_0_ of 5.4 eV to lower energy and a less intense whiteline feature compared to that of the [Fe­(acac)_2_(BA)_2_] complex, suggesting a change in the nature of the coordinating atoms around Fe. Since sulfur is less electronegative than oxygen, a higher electron density around Fe is expected, lowering the absorption edge energy (cf. Figure S11). Accordingly, *in situ* PXRD identifies FeS as a crystalline intermediate prior to the formation of Fe_3_S_4_ as the final product. Therefore, we assign the third component to the crystalline intermediate FeS (mackinawite), in which Fe^2+^ is sulfur-coordinated in a tetrahedral symmetry. The last component (displayed in red) corresponds to Fe_3_S_4_ with an E_0_ at 7118.8 eV versus 7117.8 eV for FeS, thereby evidencing the partial oxidation to Fe^3+^. To further corroborate these assignments, we compare both recovered spectra of FeS (Figure 4e) and Fe_3_S_4_ (Figure 4f) with their corresponding theoretical XANES spectra from FEFF calculations, evidencing good agreement between experiment and theory. Herein, we highlight that the *in situ* characterization enables us to capture the true electronic structure of FeS (mackinawite), avoiding any misleading effects of air-induced restructuring. These effects have likely contributed to misinterpretations in prior XANES studies.^51^

In the bottom part of Figure 4c, we depict the relative concentration of the four identified components over time. Initially, Fe­(acac)_3_ reduces to the [Fe­(acac)_2_(BA)_2_] complex immediately upon heating the reaction. The molecular intermediate remains as the predominant species in solution until around 110 °C (∼87 min), when the formation of the metastable intermediate FeS (mackinawite) starts taking place. At ∼ 165 °C (150 min), the fraction of FeS in solution reaches 100% and then starts decreasing with the concomitant increase in the fraction of Fe_3_S_4_ (greigite). It is clear that Fe_3_S_4_ only appears once the contribution of FeS starts decreasing, ruling out parallel reactions and confirming the topotactic transformation. Finally, Fe_3_S_4_ becomes the dominant constituent from 185 min reaction time, *i.e*., after 24 min at 180 °C.

As a proof of concept, we demonstrate the use of vtc-XES as an effective tool for real-time monitoring of ligand coordination changes around Fe in solution and at high temperature, complementing the findings from HERFD-XANES. As shown in Figure 4b, vtc-XES probes transitions from ligand n*p* and n*s* orbitals to the Fe 1*s* core hole, giving rise to the Kβ_2,5_ and Kβ’’ lines, respectively.^52,53^ The *in situ* vtc-XES dataset (Figure 4g) reveals that the Kβ_2,5_ line initially splits into two features, which evolves into a single peak as the reaction progresses, suggesting a change in the Fe-ligand bonding environment.^52^ The first spectrum matches well with the previously reported experimental features for Fe­(acac)_3_ in which the Kβ’’ line is absent,^35,36^ as well as with our DFT calculations shown in Figure S12. The two features at ∼ 7106.5 eV and ∼ 7111.5 eV originate from the π-bonding and π-antibonding orbitals, respectively.^37,54^

To visualize the reaction evolution, in Figure 4h, we extract the vtc-XES spectra for each one of the four compounds in the reaction at the time point corresponding to its maximum concentration (cf. Figure 4c, bottom). The attempt to employ MCR-ALS analysis on the vtc-XES dataset generated nonreliable results, due to the limited number of spectra. The spectra of both Fe­(acac)_3_ and [Fe­(acac)_2_(BA)_2_] exhibit a double peak in the Kβ_2,5_ line, showing good agreement between experiment and theory (Figure S12). Despite the similarity between the two spectra, we observe the emergence of the Kβ’’ line at ∼ 7091.5 eV for the intermediate [Fe­(acac)_2_(BA)_2_] complex (cf. Figure S13), which is assigned to the O 2*s* to Fe 1*s* decay. Finally, the formation of the iron sulfide species (Fe_*x*_S_*y*_) is evidenced by the emergence of the single feature for the Kβ_2,5_ line at around 7109 eV. Notably, the vtc-XES spectra of FeS (mackinawite) and Fe_3_S_4_ (greigite) exhibit the satellite Kβ’’ line at 7099.5–7100.0 eV, which is slightly more intense and shifted by approximately 8 eV to higher energy compared to the Kβ’’ feature in the [Fe­(acac)_2_(BA)_2_] complex. These observations reveal the change in the ligand from O­(2*s*) to S­(3*s*) when evolving from molecular intermediate to the Fe_*x*_S_*y*_ species, in line with theoretical predictions.^36^ Similarly, the comparison of the final product Fe_3_S_4_ with Fe_3_O_4_, used as a reference for its crystalline oxide analogue, indicates that the Kβ_2,5_ line is much broader for Fe_3_O_4_ than for Fe_3_S_4_, while the energy position of Kβ’’ line also changes significantly, 7099.5 eV for Fe_3_S_4_ and 7093.5 eV for Fe_3_O_4_, due to the increased covalency of S­(3*s*) compared with O­(2*s*) ligands.^36,37,55^ See Supplementary Notes 3 and Figure S14, for more details.

Therefore, we show that *in situ* vtc-XES effectively validates the evolution of Fe-ligand coordination during the Fe_3_S_4_ synthesis inferred from HERFD-XANES, particularly capturing ligand-specific features such as the Kβ’’ shift upon O- to S-coordination exchange. Additionally, these distinct Kβ’’ signatures provide a reliable spectroscopic marker, in particular, for *ex situ* identification of metastable FeS (mackinawite), which is prone to oxidation to γ-FeO­(OH) (lepidocrocite) upon ambient exposure.^56^ This enables an unambiguous assessment of whether structural and electronic changes arise from air exposure or are intrinsic effects related to nanoscale dimensions or amorphous character.

### Insights into the Local Environment and Oxidation State Using Pre-Edge Features

To gain further insights into the local environment, oxidation state, and crystal-field splitting of the components during the synthesis of Fe_3_S_4_ nanosheets, we analyze the pre-edge region (1*s*→3*d* transition), shown in Figure 5a. Fitting the pre-edge peaks of the four compounds recovered by the MCR-ALS method allows us to assign the origin of each electronic transition contributing to these features. Figure 5b shows the energy diagram of the *d* orbital splitting into different levels due to the surrounding ligand field: t_2g_ and e_g_ for O_h_ symmetry, and e and t_2_ in *T*_d_ symmetry.

Figure 5c displays the pre-edge of the initial state of the reaction (i.e., Fe­(acac)_3_), with a centroid at 7113.38 eV, which can be fitted into two components at 7112.78 and 7114.34 eV. These two features correspond to the ^4^T_2g_ and ^4^E_g_ final states, respectively, which are generated by the $|t_{2g}^{4}e_{g}^{2}\rangle$ and $|t_{2g}^{3}e_{g}^{3}\rangle$ excited states. The peak energy difference between the two fitted peaks provides the crystal-field splitting parameter (Δ_oct_), yielding an experimental value of 1.56 eV. Additionally, the intensity ratio between the two features is 1.56, which agrees well with the reported ratio of 1.5 for Fe­(acac)_3_.^42^ These results are predicted by the crystal-field theory for octahedral high-spin Fe^3+^ complexes, such as Fe­(acac)_3._ ^42,57^ See Supplementary Notes 4 and Figure S15 for reference of the excited configuration that generate each final state.^58,59^

The pre-edge of the final product Fe_3_S_4_ has the centroid at 7113.26 eV, as shown in Figure 5d. The pre-edge can be fitted with three components at 7111.91, 7113.01, and 7114.58 eV, which originate from the different coordination and oxidation states of Fe ions in the cubic spinel structure of Fe_3_S_4_. Although similar findings were previously reported for the Fe_3_O_4_,^60,61^ assigning the origin of each feature is nontrivial. We assume that the most intense peak at 7113.01 eV originates from the Fe^3+^ in *T*_d_ symmetry (final state ^5^T_2_), which corresponds to transitions that are nominally dipole forbidden, but become partially allowed due to symmetry considerations. The feature at 7111.91 eV results from the ^4^T_1g_ final state from Fe^2+^ in O_h_ sites, while the peak at 7114.58 eV can be assigned to the ^5^E_g_ final state from Fe^3+^ in O_h_ sites. However, a small contribution of the second ^4^T_1g_ final state from Fe^2+^ in O_h_ sites cannot be ruled out. The pre-edge fitting of the recovered spectra by MCR-ALS analysis for Fe­(acac)_3_ and Fe_3_S_4_ exhibits features consistent to those derived from the RIXS maps (Figure S16), confirming the reliability of the fit.

The pre-edge fitting of the molecular intermediate [Fe­(acac)_2_(BA)_2_] is given in Figure 5e. The centroid at 7112.54 eV, compared to 7113.38 eV for Fe­(acac)_3_, indicates the reduction to Fe^2+^. However, three features are resolved, as predicted by crystal-field theory.^41,42,57^ The $|t_{2g}^{5}e_{g}^{2}\rangle$ excited configuration generates the ^4^T_1g_ final state observed at 7111.96 eV, while the $|t_{2g}^{4}e_{g}^{3}\rangle$ excited configuration produces the ^4^T_2g_ and ^4^T_1g_ states at 7113.20 and 7114.46 eV, respectively. Δ_oct_ is estimated as 1.24 eV from the energy difference between the lower-energy ^4^T_1g_ state and the ^4^T_2g_ state, which is consistent with values reported for Fe^2+^ complexes with ligands of similar field strength in O_h_ symmetry,^42^ corroborating the structure of the proposed molecular complex.

Finally, we fit the pre-edge of the crystalline intermediate FeS (mackinawite) with two contributions: at 7112.11 and 7113.50 eV (Figure 5f). The first feature corresponds to overlapping transitions of several unresolved final states, including ^4^A_2_, ^4^T_2_, and ^4^T_1_ states, while the higher-energy peak is primarily assigned to the ^4^T_1_ state.^41,62^ Similar observations have been reported for the pre-edge features of minerals containing Fe^2+^ in a *T*_d_ symmetry.^41,60^ The ^4^T_2_ and both ^4^T_1_ states enhance the intensity of the pre-edge feature due to dipole-allowed transitions. Additionally, the centroid of the pre-edge appears at a lower energy compared to that of [Fe­(acac)_2_(BA)_2_], in line with a change in coordination environment from Fe–O to Fe–S while retaining the Fe^2+^ oxidation state. Yet, the centroid is at a lower energy than that of the Fe_3_S_4_ spectrum reflecting the partial reduction of Fe^3+^ to Fe^2+^ in a similar sulfur-coordinating environment. Table S5 presents the results of the pre-edge fit with pseudo-Voigt functions for the recovered spectra from the MCR-ALS analysis.

The comparison of the pre-edge features of the four MCR-ALS recovered components reveals changes in oxidation state, symmetry, and coordination environment throughout the reaction. This demonstrates that pre-edge fitting can also be carried out for the recovered species from *in situ* reactions, enabling the analysis of reaction intermediates that cannot be isolated and assessed *ex situ*.

## Conclusions

In this study, we demonstrate the advantages of multimodal *in situ* X-ray methods for elucidating the complex reaction mechanism leading to the formation of crumpled Fe_3_S_4_ nanosheets. By using *in situ* PXRD, we discover FeS (mackinawite) as a reaction intermediate that grows preferentially along the [001] axis, facilitated by its layered structure held together by weak van der Waals forces, leading to the formation of crumpled thin nanosheets. The subsequent conversion to Fe_3_S_4_ (greigite) takes place via a solid-state structural reorganization, allowing the material to partially retain its initial morphology, resulting in an intermediate-imposed shape.

*In situ* HERFD-XANES enables tracking the electronic structure throughout the reaction. By coupling experimental data with theoretical calculations, we propose that upon heating, the Fe­(acac)_3_ precursor rapidly reduces and coordinates with solvent molecules to form the intermediate [Fe­(acac)_2_(BA)_2_] complex, which then transforms into FeS before further converting to Fe_3_S_4_. The high resolution of the method, combined with the *in situ* setup, enables accurate distinction between FeS and Fe_3_S_4_ by resolving the variations in the pre-edge position and intensity, and by capturing characteristic differences across the near-edge region. This also allows us to capture the true electronic structure of the metastable FeS, without artifacts from air-induced restructuring. Notably, our findings also demonstrate that *in situ* vtc-XES can monitor changes in ligand coordination dynamics during high temperature solvothermal reactions, providing distinct signatures for O- and S-coordination.

In summary, our findings provide fundamental insights into how synthesis pathways dictate nanoparticle morphology and electronic structure. These results not only deepen our understanding of Fe–S chemistry but also highlight the potential of applying such *in situ* approaches to geological, mineralogical, and materials science problems where metastable phases and morphology control are critical.

## Experimental Section

### Chemicals

Iron acetylacetonate­(III) (Fe­(acac)_3_, ≥ 99.9% trace metals basis), and benzyl alcohol (BA, ≥ 99.0%) were purchased from Sigma-Aldrich, and thioacetamide (TAA, 98%) was obtained from Thermo Scientific. The chemicals were used as received without further purification.

### Synthesis of Crumpled Fe_3_S_4_ Nanosheets

The Fe_3_S_4_ nanosheet-like structures were synthesized via a solvothermal approach. In separate containers, a 0.2 mol/L solution of Fe­(acac)_3_ in benzyl alcohol (BA) and a 0.6 mol/L solution of thioacetamide in benzyl alcohol were prepared at ambient conditions. Both solutions were homogeneously mixed, and an aliquot of 200 μL was transferred to a polyether ether ketone—PEEK reaction container, which was sealed and heated using our homemade reaction cell for *in situ* experiments.^31,33^ The solutions were heated at 180 °C under constant magnetic stirring for 60 min at a heating ramp of 10 °C/min, or 1 °C/min. For comparison purposes, the reaction was also carried out at 140 and 160 °C. For *ex situ* characterizations, the precipitate was washed four times with BA and five times with ethanol, and dried at room temperature.

### Characterization

#### S­(T)­EM

The scanning electron microscopy (SEM) and scanning transmission electron microscopy (STEM) images were acquired using a Regulus 8220 microscope (Hitachi High Technologies Corp., Japan) at an acceleration voltage of 30 kV.

#### TEM

The transmission electron microscopy (TEM) and high-resolution TEM (HRTEM) images, selected area electron diffraction (SAED) patterns, and energy dispersive X-ray spectroscopy (EDX) measurements were collected with a JEM-2200FS microscope (JEOL Ltd., Japan) operated at an acceleration voltage of 200 kV.

#### Thermogravimetric Analysis

The thermogravimetric analysis (TGA) was conducted using a Discovery SDT 650 thermogravimetric analyzer (TA Instruments, USA) under N_2_ flow, with a heating rate of 5 °C/min.

#### AFM

Atomic force microscopy (AFM) topography images were recorded under ambient conditions using a Multimode AFM (Bruker Corp., USA; formerly Digital Instruments) with a Nanoscope III controller plus phase extender. The data shown here were acquired with a silicon cantilever (PPPFMR, Nanosensors; resonance frequency *f*_R_ ≈ 69 kHz, Q-factor Q ≈ 200, spring constant *c*_*z*_ ≈ 2.4 N/m) operated in tapping mode. For image representation, a background subtraction was performed using WSxM software.^63^ Samples for AFM imaging were prepared by dispersing a small amount of powder in ethanol using ultrasonication for 1 h. The resulting dispersion was drop-cast onto a Si wafer (0.5 cm × 0.5 cm), and dried under ambient conditions.

#### Magnetic Measurements

The magnetic properties of the nanoparticles were characterized using a Physical Property Measurement System (PPMS, Quantum Design) in the Vibrating Sample Magnetometer configuration (VSM). ± 3T hysteresis measurements were recorded at 300 and 5 K after field cooling at 3 T to probe the presence of an oxide shell. Nanoparticles were studied in powder state, placing about 10 mg in a polypropylene holder under inert atmosphere. Zero-field cooling/Field cooling (ZFC/FC) measurements were performed keeping an external magnetic field of 5 mT and ranging the temperature between 5 and 300 K.

### X-ray Methods

#### PXRD

PXRD measurements were carried out at beamline P21.1 of PETRA III at Deutsches Elektronen-Synchrotron (DESY), Hamburg, Germany.^64^ Diffraction patterns were collected every second at an incident X-ray energy of 101.84 keV (λ of 0.12183 Å) employing a flat panel detector XRD1621 (PerkinElmer Inc., USA) with a pixel size of 200 × 200 μm^2^ and a sample-to-detector distance (SDD) of 1.54 m. The SDD value was obtained from the calibration from the measurement of the LaB_6_ powder filled in the glass reaction container of the cell for *in situ* reactions.

#### HERFD-XANES and vtc-XES

All spectra were acquired at beamline ID26 of the European Synchrotron Radiation Facility (ESRF), Grenoble, France, employing a Si(111) double-crystal monochromator (DCM). For HERFD-XANES, the incident energy was varied from 7.10 to 7.22 keV over the Fe K-edge, and the maximum of the Fe Kα_1_ line was measured using an emission spectrometer equipped with a Ge(440) analyzer crystal aligned in a Rowland geometry of 1 m radius. The incident energy was calibrated by setting the maximum of the first derivative of the transmission of an Fe foil to 7.112 keV, which was measured in ultra-high vacuum. The emission energy was calibrated using the maximum of the Fe Kβ emission at 7.058 keV. HERFD-XANES data were recorded in continuous mode every 35 s with a step size of 0.1 eV. To eliminate radiation damage, multiple spots were measured and the beam position was shifted on the reaction cell after each scan during the reactions. The RIXS maps were acquired from 7.108 to 7.118 keV in the incident energy and from 6.380 to 6.410 keV in the emission energy range for the initial state and the final product of the reaction in solution.

vtc-XES spectra were measured in the energy range of 7.065 to 7.135 keV using four Ge(620) crystal analyzers positioned in a Rowland arrangement. To enable data collection at relevant time scales during *in situ* measurements, data was collected in 2 eV steps with a 1 s acquisition time per point, except in the range of 7.082 to 7.125 keV. In this region, the energy step size was decreased to 0.5 eV and the acquisition time increased to 3 s per point to ensure higher resolution. Taking into account the motors’ movements, each vtc-XES spectrum was collected in 490 s.

### Data Processing and Simulation

#### PXRD

The 2D diffraction patterns were integrated using pyFAI,^65^ which was used for obtaining instrumental parameters, masking unwanted elements such as dead pixels, shadows from the beam stop, etc. For the background subtraction, only thioacetamide dissolved in benzyl alcohol was measured under the same conditions of the synthesis, i.e., at 180 °C at a heating rate of 10 °C/min. The data were averaged over 60 frames, yielding a time resolution of 60 s. The resulting PXRD patterns were sequentially refined using GSAS-II package,^66^ starting from the PXRD pattern at the end of the reaction and moving back to earlier reaction times. The refinements were performed using the crystallographic data of cubic spinel Fe_3_S_4_ (*Fd3̅m* (227) space group) from the Inorganic Crystal Structure Database (ICSD)-42535, and tetragonal FeS (*P4/nmm* (129) space group) ICSD-81087.

#### HERFD-XANES and vtc-XES

The data processing was carried out in Python with a self-written code. For the HERFD-XANES data, the position and normalization of the edge jump were determined with LARCH-XAFS module.^67^ The spectra were smoothed using a Savitzky–Golay filter. A 1.64 eV window length (≡ 25 data points) was used for HERFD-XANES, and a 7.64 eV window length (≡ 9 data points) was used for vtc-XES, both with the same second-order polynomial. The data were further processed with the NumPy and SciPy packages.^68,69^ Theoretical simulations of the XANES and vtc-XES spectra for the macroscopic crystals were calculated using FEFF09,^55,70^ with settings listed in Supplementary Table S6. The absolute energies of the calculated spectra were aligned with experimental data using the SETEDGE command. For the molecular iron complexes, the XANES and vtc-XES simulations were performed using the ORCA 5.0.4 code,^71^ and the settings are given in Table S7. Avogadro version 1.2.0 was employed for building the molecular complexes for DFT optimization.^72^ The position and intensity of the pre-edge were determined with least-squared fitting algorithm, where the features were fitted by pseudo-Voigt profiles.

## Supplementary Material



## Data Availability

All data generated and analyzed in this study are available in the ZFDM Repository, University of Hamburg, at 10.25592/uhhfdm.17911.

## Notes

Additional characterization including TEM, S­(T)­EM, AFM, and TG; magnetic property measurements; supplementary Rietveld refinement results; notes and fitting parameters for the MCR-ALS analysis; DFT calculations; notes on vtc-XES data of Fe_3_O_4_; complementary detailed information on pre-edge fitting; and settings used for FEFF and DFT calculations (PDF)
