# Supplementary material for: In situ X‑ray Synchrotron Studies Reveal the Nucleation and Topotactic Transformation of Iron Sulfide Nanosheets
Source: J Am Chem Soc. 2025 Dec 12;147(51):47409–20. doi: 10.1021/jacs.5c15843 (PMC12752459; doi:10.1021/jacs.5c15843)
Supplement: Supplementary file 1 [file ja5c15843_si_001.pdf]

SUPPORTING INFORMATION

In situ X-ray synchrotron studies reveal the nucleation and topotactic transformation of iron sulfide nanosheets

Cecilia A. Zito,[1,2,#] Lars Klemeyer,[1,2,#] Francesco Caddeo,[1] Brian Jessen,[1] Sani Y. Harouna-Mayer,[1,2] Lise-Marie Lacroix,[3,4] Malte Langfeldt,[1] Tjark L. R. Gröne,[1] Jagadesh K. Kesavan,[1,2] Chia-Shuo Hsu,[1] Alexander Schwarz,[5] Ann-Christin Dippel,[6] Fernando Igoa Saldaña,[6] Blanka Detlefs,[7] Dorota Koziej[1,2*]

[1] University of Hamburg, Institute for Nanostructure and Solid-State Physics, Center for Hybrid Nanostructures, Luruper Chaussee 149, 22761 Hamburg, Germany

[2] The Hamburg Center for Ultrafast Imaging, 22761 Hamburg, Germany

[3] Laboratoire de Physique et Chimie des Nano-Objets UMR 5215 INSA, CNRS, UPS, Université de Toulouse, 135 avenue de Rangueil, F-31077 Toulouse cedex 4, France.

[4] Institut Universitaire de France (IUF), 103 boulevard Saint Michel, 75005 Paris, France

[5] University of Hamburg, Institute for Nanostructure and Solid-State Physics, Jungiusstraße 11a, 20355 Hamburg, Germany

[6] Deutsches Elektronen-Synchrotron DESY, Notkestraße 85, 22607 Hamburg, Germany.

[7] ESRF, The European Synchrotron Facility, 71 Avenue des Martyrs, CS40220, 38043 Grenoble Cedex 9, France

*Corresponding author: dorota.koziej@uni-hamburg.de

# These authors contributed equally

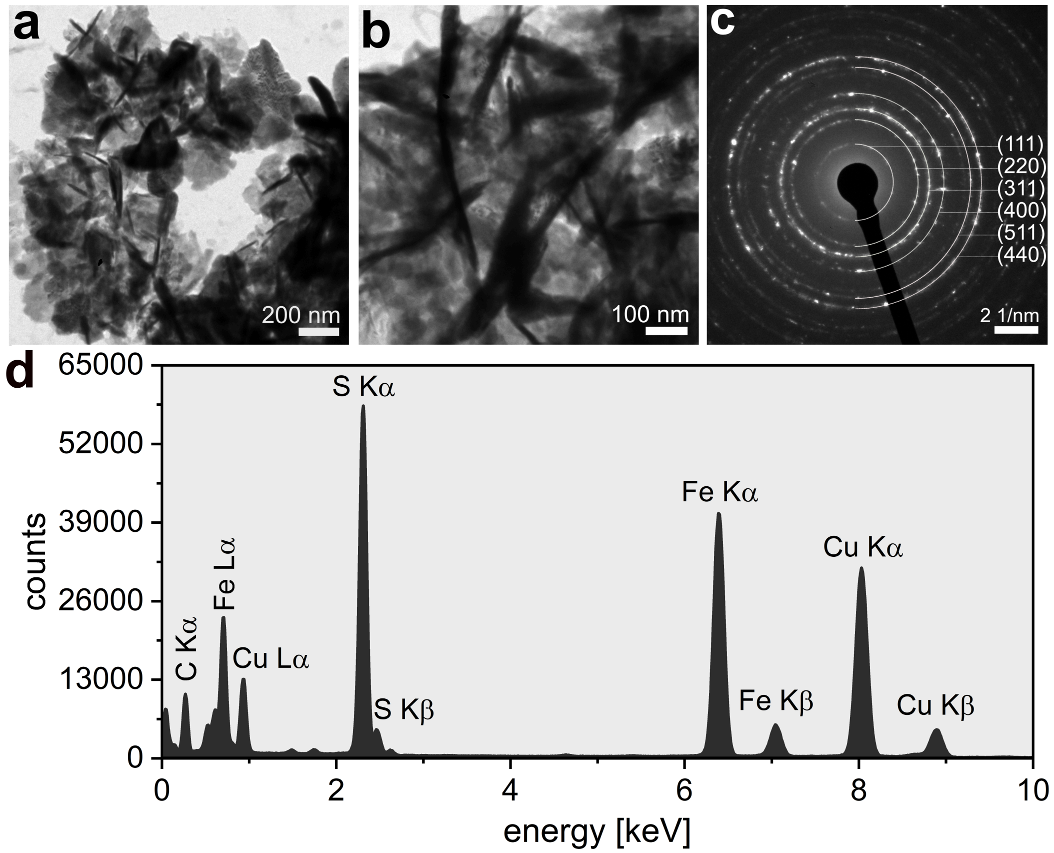

Figure S1. **Additional TEM characterization of Fe_3S_4 nanosheets. (a)** TEM image with the overview of the Fe_3S_4 nanosheet-like structures. **(b)** TEM image corresponding to the area for the SAED analysis. **(c)** SAED pattern, with diffraction rings indexed to the planes of Fe_3S_4. **(d)** EDX spectrum with contributions from all elements in the sample. The semi-quantitative EDX analysis, considering the relative intensities of K lines of Fe and S, yields an approximate atomic ratio S:Fe of 1.35, which is consistent with the empirical value of 1.33.

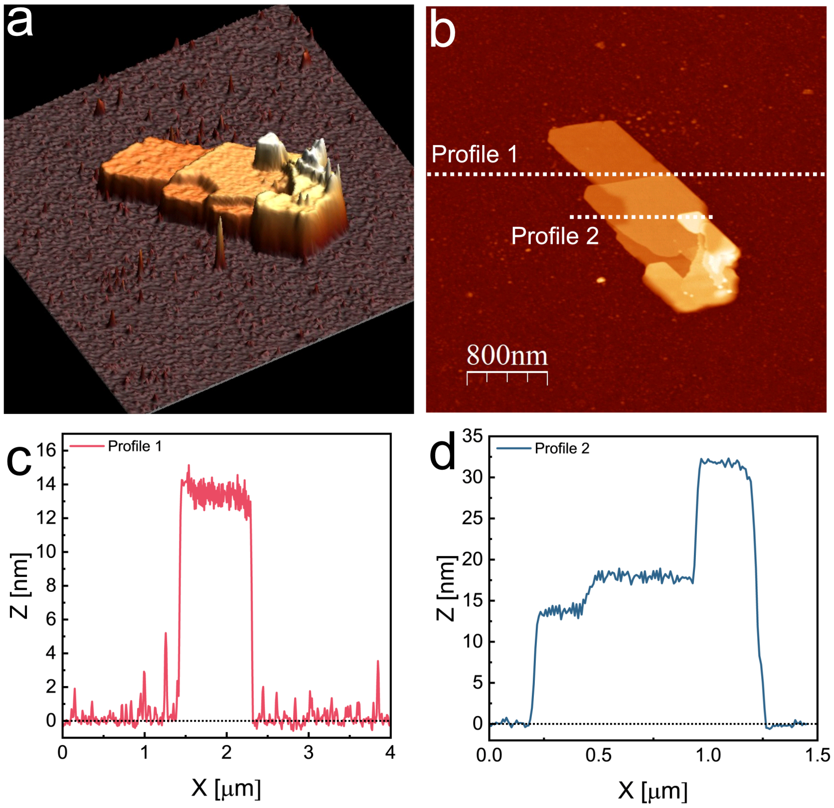

Figure S2. **AFM characterization of Fe_3S_4 nanosheets. (a)** Three-dimensional perspective view of the Fe_3S_4 nanosheet. **(b)** AFM image of an individual nanosheet, with two different regions selected for the height measurements. **(c,d)** Corresponding height profile along the regions delimited in (b), showing a thicknesses of ~14 nm,~18 nm, and ~32 nm.

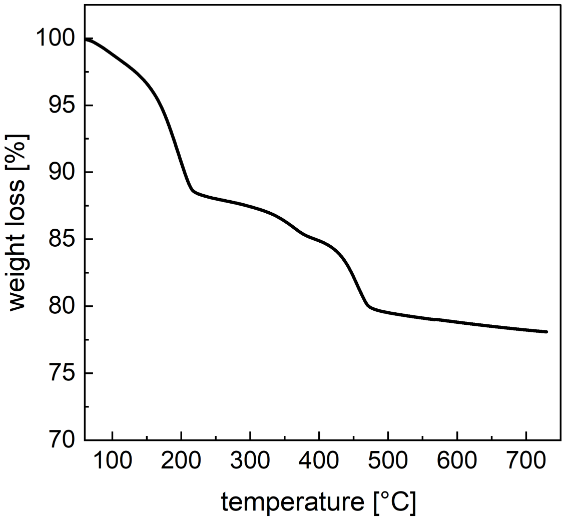

Figure S3. Thermogravimetric analysis (TG) of the Fe_3S_4 nanosheet-like structures. The curve shows a weight loss of ~12.6% up to 300 °C corresponding to residual organic contribution in the sample. The subsequent weight loss up to *ca*. 480 °C is assigned to the phase transformation of Fe_3S_4 to Fe_xS_y, with x,y ≈ 1.

Supplementary Notes 1: Magnetic properties

To further characterize the synthesized Fe_3S_4 nanosheet-like structures, we assess their magnetic behavior. In the magnetization curves measured at 5 K and 300 K (**Figure S4a,b**), we observe hysteresis for both temperatures, characterized by coercive fields of 100 mT and 30 mT, respectively. These values are in agreement with the reported literature.[1, 2] The magnetization curve at 5 K was recorded after cooling the sample from room temperature under an external field of +3T. This procedure allows us to probe the presence of an oxide shell, which would induce an exchange bias on the magnetization curve, shifting the hysteresis cycle towards the negative fields.[3] Here, the hysteresis cycle is symmetric and centered around 0, discarding any oxide shell at the surface of the nanoparticles. However, the constant increase of magnetization as a function of magnetic field reveals a paramagnetic contribution, which can be due to spin canting at the surface of the nanoparticles, as commonly observed.[4] As shown in **Figure S4c**, the thermal evolution of the magnetization during the zero-field cooling (ZFC)/field cooling (FC) measurement confirms that the Fe_3S_4 nanosheets exhibit a ferrimagnetic behavior at room temperature, as expected.[5] The blocking temperature (T_B), normally revealed by the maximum of the ZFC curve, exceeds 300 K and thus could not be determined. This is in agreement with the fairly large size of the Fe_3S_4 nanostructures observed. For example, a T_B of 180 K was reported for 10 to 20 nm large Fe_3S_4 nanoplatelets,[2] while sphere-like nanoparticles above 16 nm exhibit T_B>300K.[1] The flatness of the FC curve in **Figure S4c** reveals a strong interaction between the nanoparticles. From **Figure S4b**, we determine the saturation magnetization (M_s) to be 28.9 Am^2/kg at 5 K, which is in line with, or even larger than, most of the experimentally reported values.[2, 6, 7] Nevertheless, it remains lower than the bulk values, which vary between 43 and 65 Am^2/kg, depending on the evaluation method.[5, 6, 8] The summary of the magnetic properties is given in **Table S1**.

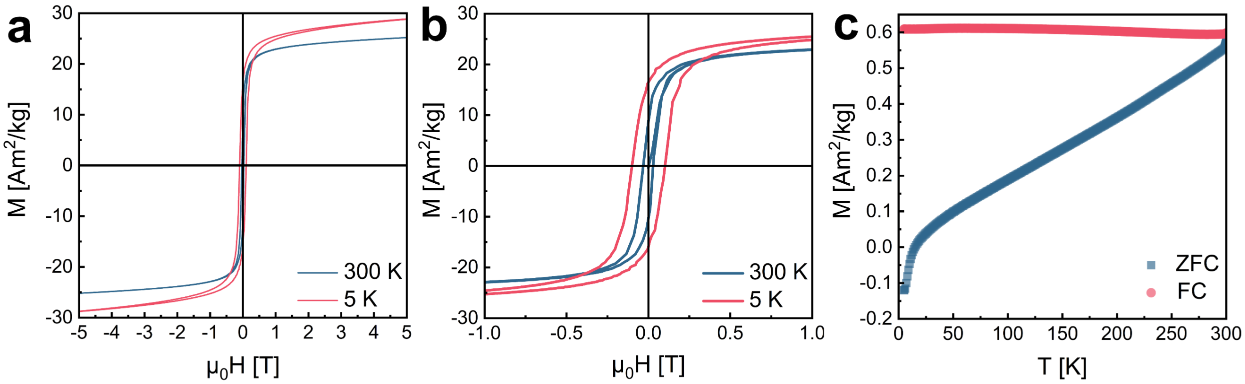

Figure S4. Magnetic properties of the Fe_3S_4 nanosheets. (a) Magnetization as a function of the applied field recorded at 300 K and 5 K after cooling down the sample under an applied magnetic field of $\mu_0H = 3T$, **(b)** Enlargement of (a) showing a narrower range of the applied field, evidencing the coercive fields. **(c)** Temperature dependence of the magnetization recorded under an external field of μ_0H = 5 mT once the sample has been cooled down in absence of magnetic field (zero field cooling, ZFC) or under a magnetic field (field cooling, FC).

Table S1: Magnetic properties of the Fe_3S_4 nanosheets. Values for the saturation magnetization (Ms), ratio between remnant magnetization (Mr) and M_s, and coercive field.

Temperature (K)	M_s (Am/kg)	M_r/M_s (Am^2/kg)	μ_0Hc (mT)
5	28.9	56%	100
300	25.2	31%	30

Table S2. Results from the Rietveld refinement at different reaction times during the synthesis of Fe_3S_4. The refined crystallite size of FeS becomes unreasonable at low fraction of FeS (from 38 min and onwards) due to the limitation of the technique. Reaction times of 10, 13, 14, 15 min correspond to temperatures of 138 °C, 168 °C, 178 °C, and 184 °C respectively, whereas longer reaction times correspond to 180 °C.

Time (min)	Fe_3S_4 weight fraction (%)	FeS weight fraction (%)	Size Fe_3S_4 (nm)	Axial size FeS (nm)	Equatorial size FeS (nm)	Goodness of fit - Rw (%)
10	0	100	-	1	13	51.3
13	3	97	14.3	3	72	50.7
14	5	95	15	3	37	40.8
15	24	76	17.1	3	55	45.4
16	38	62	18.3	4	40	36.9
18	53	47	17.5	3	37	29.6
19	58	42	16.8	3	51	29.9
20	62	38	18.2	3	56	29.8
21	65	35	16.8	3	58	30.1
23	67	33	17.1	2	63	27.3
25	68	32	18.5	2	55	37.2
26	74	26	17.8	2	112	25.6
27	76	24	17.4	2	87	25.0
30	82	18	17.9	3	94	24.6
34	87	13	18.6	3	62	24.6
36	91	9	19.4	3	54	23.8
38	90	10	20	2	780	24.9
39	94	6	19.8	3	1000	22.6
41	94	6	19.8	3	1000	21.9
42	95	5	19.8	2	1000	21.2
44	94	6	20	2	1000	20.8
45	97	3	20.3	3	1000	24.4
47	100	0	20.8	-	-	28.4
49	100	0	20.2	-	-	25.8
52	100	0	21.3	-	-	35.3
55	100	0	21.8	-	-	27.2
59	100	0	21.1	-	-	24.9
62	100	0	22.4	-	-	25.2
66	100	0	22.6	-	-	30.5
71	100	0	22.8	-	-	26.4
75	100	0	21.9	-	-	26.1

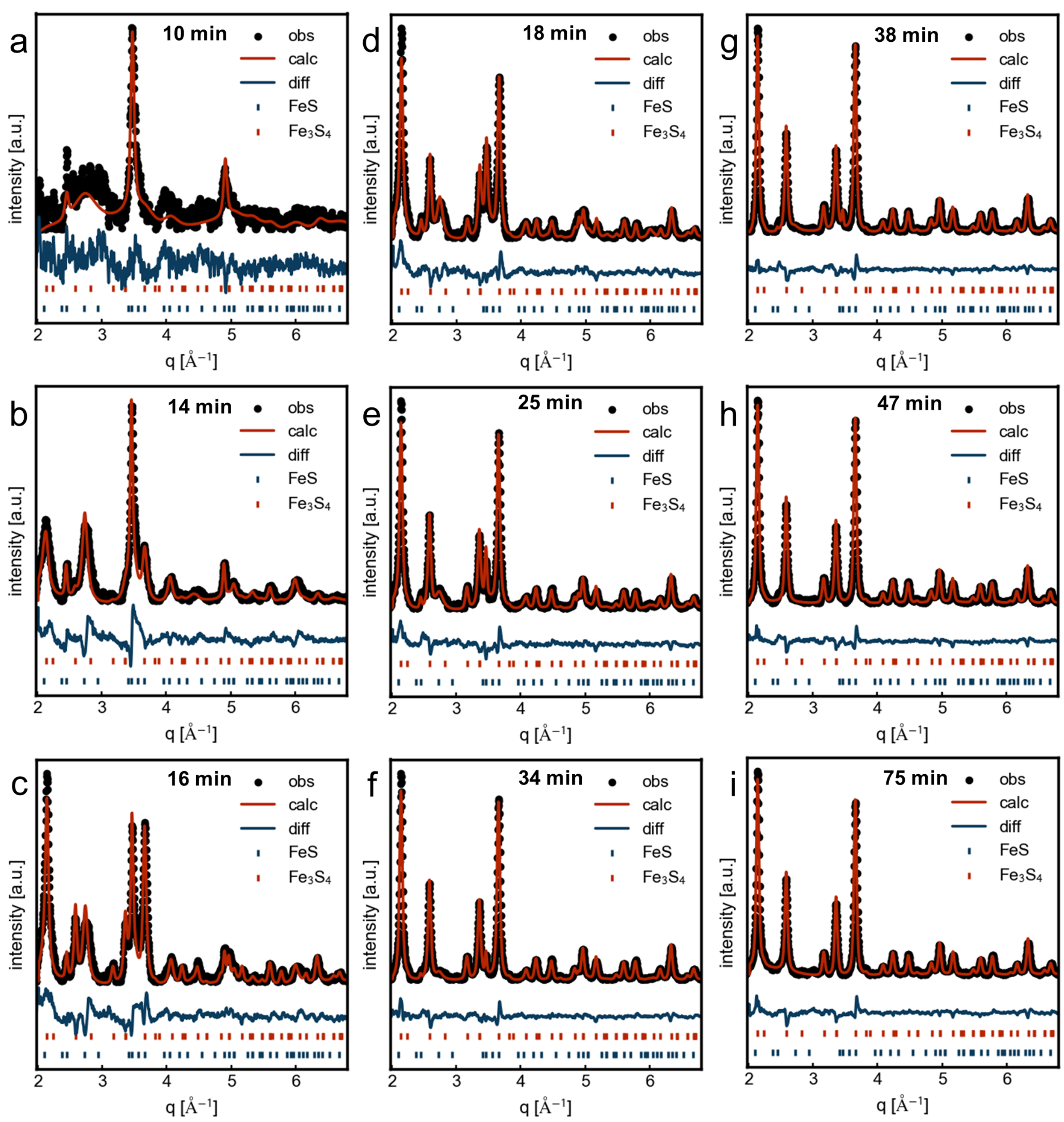

Figure S5. Sequential Rietveld refinements of the Fe_3S_4 synthesis at 180 °C at 10 °C/min at relevant time scales: **(a)** 10 min (138 °C), the first refined pattern, exhibiting the sharp and intense (200) reflection of FeS, **(b)** 14 min (178 °C), when FeS is the dominant yet poorly defined species in the reaction, **(c)** 16 min (180 °C), which shows the pronounced Fe_3S_4 reflections, **(d)** 18 min, when the FeS contribution starts decreasing considerably, **(e)** 25 min, which exhibits only small contribution of FeS, and **(f)** 34 min with a minor FeS contribution,**(g)** 38 min, when the refined contribution of FeS yields unphysical values for its crystallite size, **(h)** 47 min, when the Fe_3S_4 becomes the single crystalline phase, and **(i)** 75 min (≡ 60 min at 180 °C), corresponding to the final product.

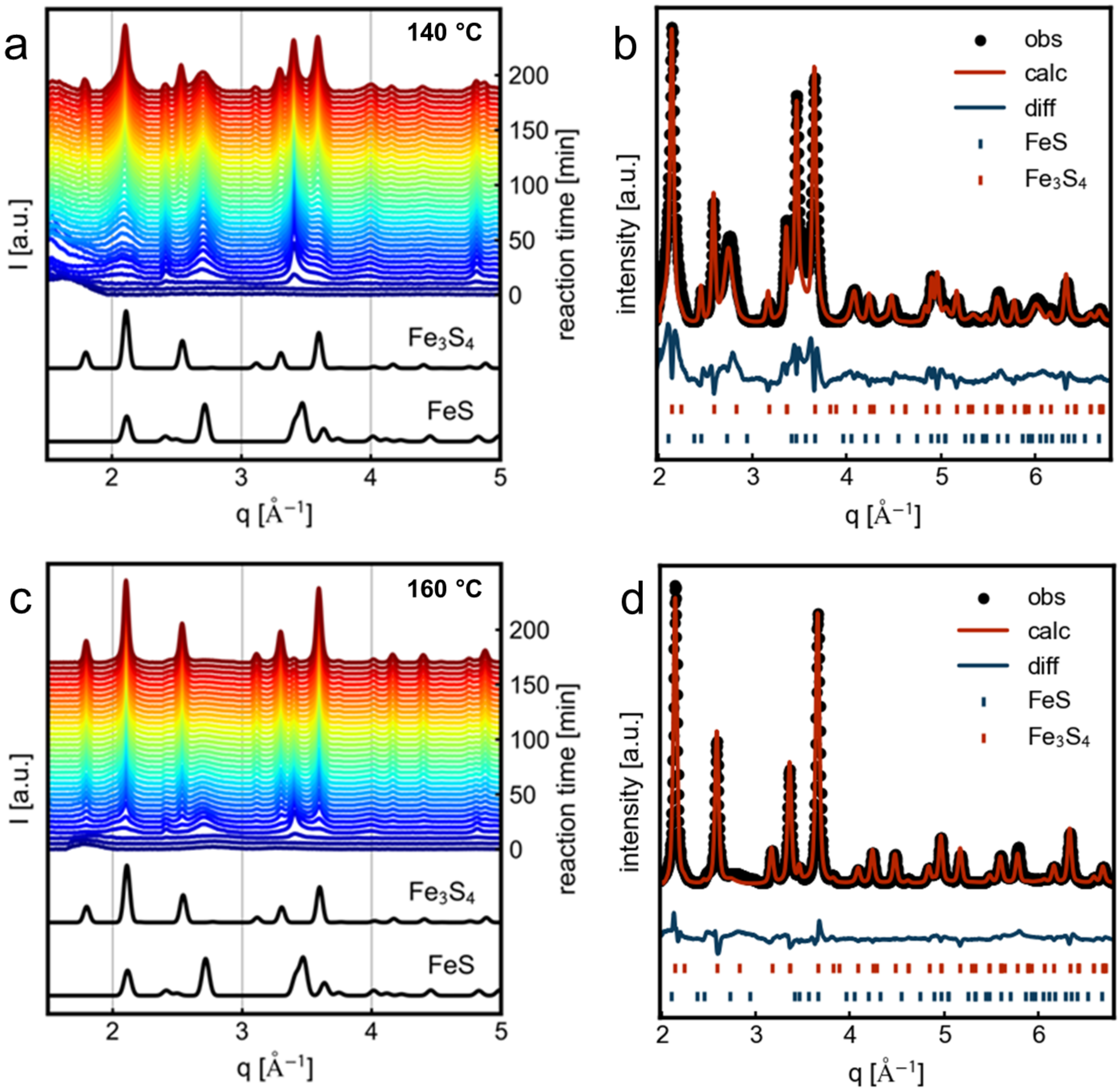

Figure S6. ***In situ*** **PXRD data for the reaction at different temperatures (140 °C and 160 °C).** **(a)** *In situ* time-resolved PXRD patterns for the synthesis at 140 °C at 10 °C/min, in which FeS mackinawite is the only observable crystalline phase at early stages of the reaction and persists by the end of the reaction along with Fe_3S_4. **(b)** Rietveld refinement of the final product in solution at 140 °C, which reveals a fraction of 61% FeS mackinawite and 39% Fe_3S_4 greigite (domain size of 32.5 nm). **(c)** *In situ* time-resolved PXRD patterns for the synthesis at 160 °C at 10 °C/min, where Fe_3S_4 becomes the predominant phase faster; however, but FeS does not fully convert to Fe_3S_4 at the end of the reaction. **(d)** Rietveld refinement of the final product in solution at 160 °C, exhibiting a fraction of 93% of Fe_3S_4 (domain size 26.8 nm) and 7% of FeS.

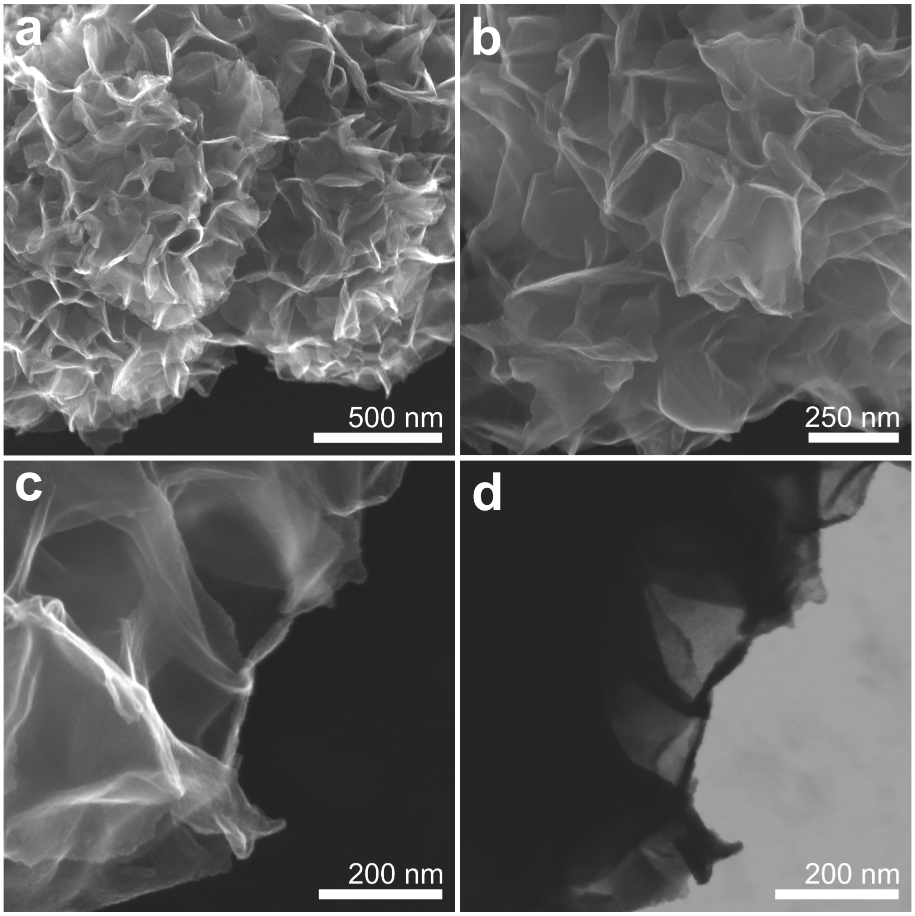

Figure S7. Morphological characterization of the product obtained at 140 °C, which is composed of 61% of FeS (mackinawite) and 39% of Fe_3S_4 (greigite). (a-c) SEM images at different magnifications, elucidating the formation of crumpled nanosheets. The interconnected thin nanosheets have a thickness of ~15 nm. **(d)** STEM image at the edge of the nanosheets, showing their thin structure.

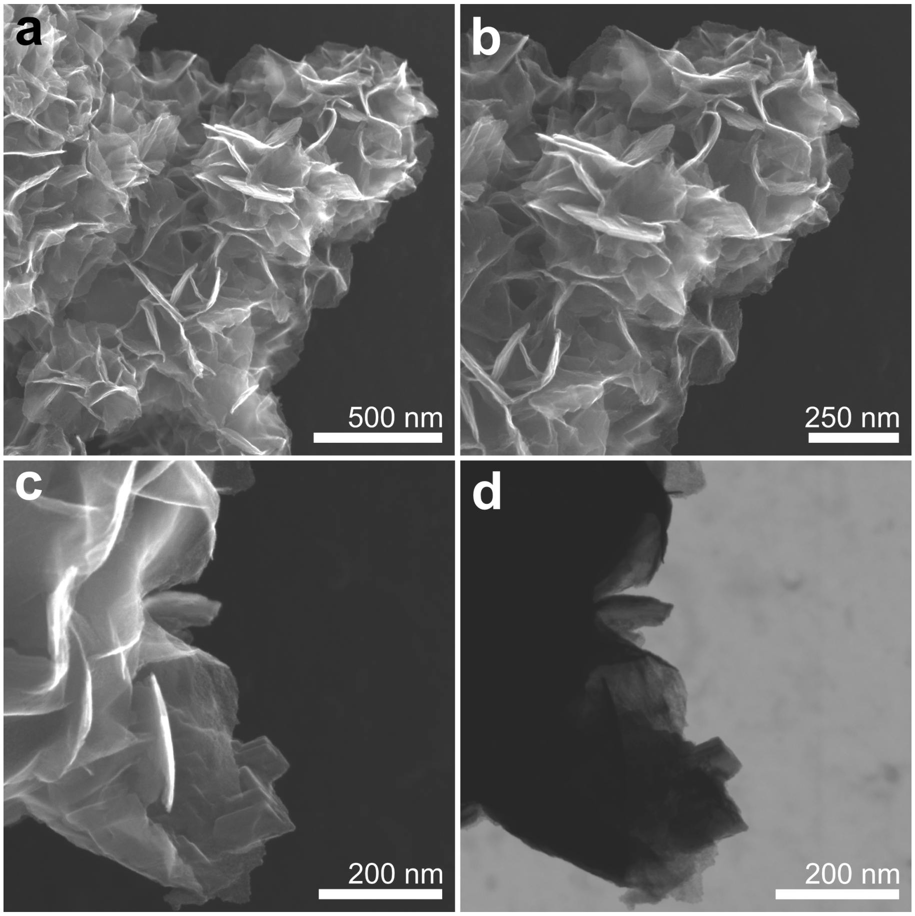

Figure S8. **Morphological characterization of the product obtained at 160 °C which is composed of 93% of Fe_3S_4 (greigite) and 7% of FeS (mackinawite). (a-c)** SEM images at different magnifications, elucidating the formation of crumpled and agglomerated nanosheets. The thickness of the interconnected thin nanosheets is around 15 nm. The nanostructures present more edges than the product obtained at 140 °C, however, the morphology is preserved when increasing the reaction temperature. **(d)** STEM image at the edge of the nanosheet-like structure.

Supplementary Notes 2: MCR-ALS analysis

Multivariate Curve Resolution by Alternating Least Squares (MCR-ALS) method allows determination of individual and independent chemical species in the *in situ* dataset. An extensive description of this analysis tool has been previously reported.[9-11]

For optimizing the analysis, the Singular Value Decomposition (SVD) results were used to determine the number of components by evaluating the eigenvalues, as displayed in **Figure S9a** and **Table S3**. For our data, we set the threshold at four components (shown in red); beyond this point, the eigenvalues align linearly, which is associated with the noise in the data. **Table S4** illustrates the fitting parameters - such as lack of fit, the variance explained and standard deviation of the residual - for the *in situ* data after MCR-ALS analysis employing four components.

To verify the possibility of additional components in our *in situ* data, we perform MCR-ALS analysis using five components (**Figure S9b**). However, it is evident that the 3rd and 5th components are highly correlated in both concentration profiles and spectral line shape, resulting in non-physically meaningful results and ruling out the existence of five components. Thus, we conclude that four independent components are present in our data set.

It is important to note that, for reliable convergence of the MCR-ALS analysis, constraints are used to ensure physically and chemically meaningful results, such as non-negativity and unimodality for the concentration and spectral features.

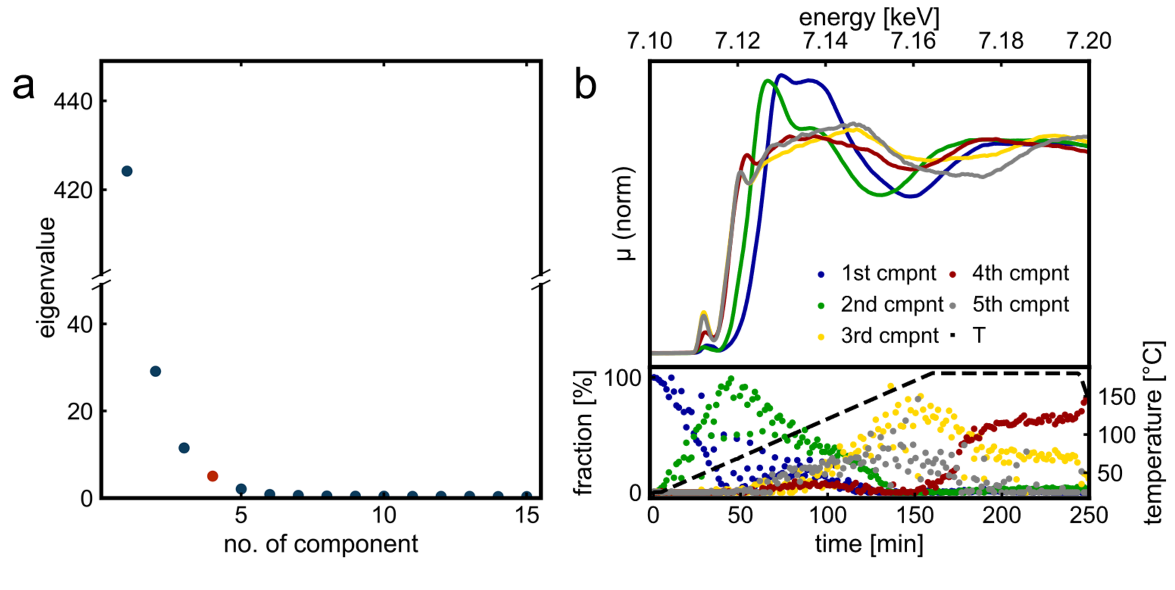

Figure S9. **MCR-ALS results for the *in situ* HERFD-XANES data set**. **(a)** Scree plot of eigenvalues with threshold at component four (red). The linearly aligned values can be associated to noise. (**b**) Recovered spectra and their relative concentration from the MCR-ALS analysis employing five components. The results are, however, unphysical as the 3rd (yellow) and 5th (grey) components highly correlated in both concentration profiles and spectral line shape.

Table S3. The first thirteen eigenvalues determined by SVD of the MCR-ALS analysis of the data. The threshold was set at four components.

Number of components	Eigenvalues
1	424.229
2	29.1556
3	11.5382
4	5.04375
5	2.07292
6	1.26235
7	0.83321
8	0.60678
9	0.46614
10	0.45054
11	0.41991
12	0.41254
13	0.38612
14	0.37665
15	0.35912
16	0.33809

Table S4. Fit quality parameters for MCR-ALS analysis for the data shown in Figure 4c.

Parameters	Value
Lack of fit (%)	0.58
R^2	99.997
σ	0.0062

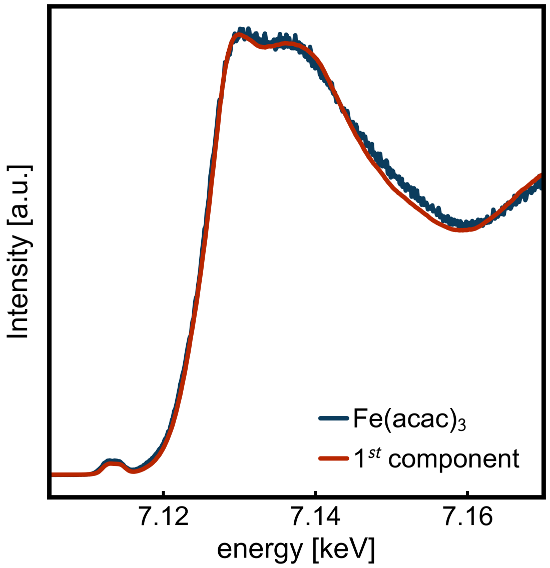

Figure S10. Identification of the starting point of the reaction. HERFD-XANES spectrum of the $Fe(acac)_3$ reference, which was diluted in BN and measured as a pellet, compared with the spectrum of the first recovered component by MCR-ALS, confirming that no major changes occur upon dissolving $Fe(acac)_3$ with thioacetamide in benzyl alcohol.

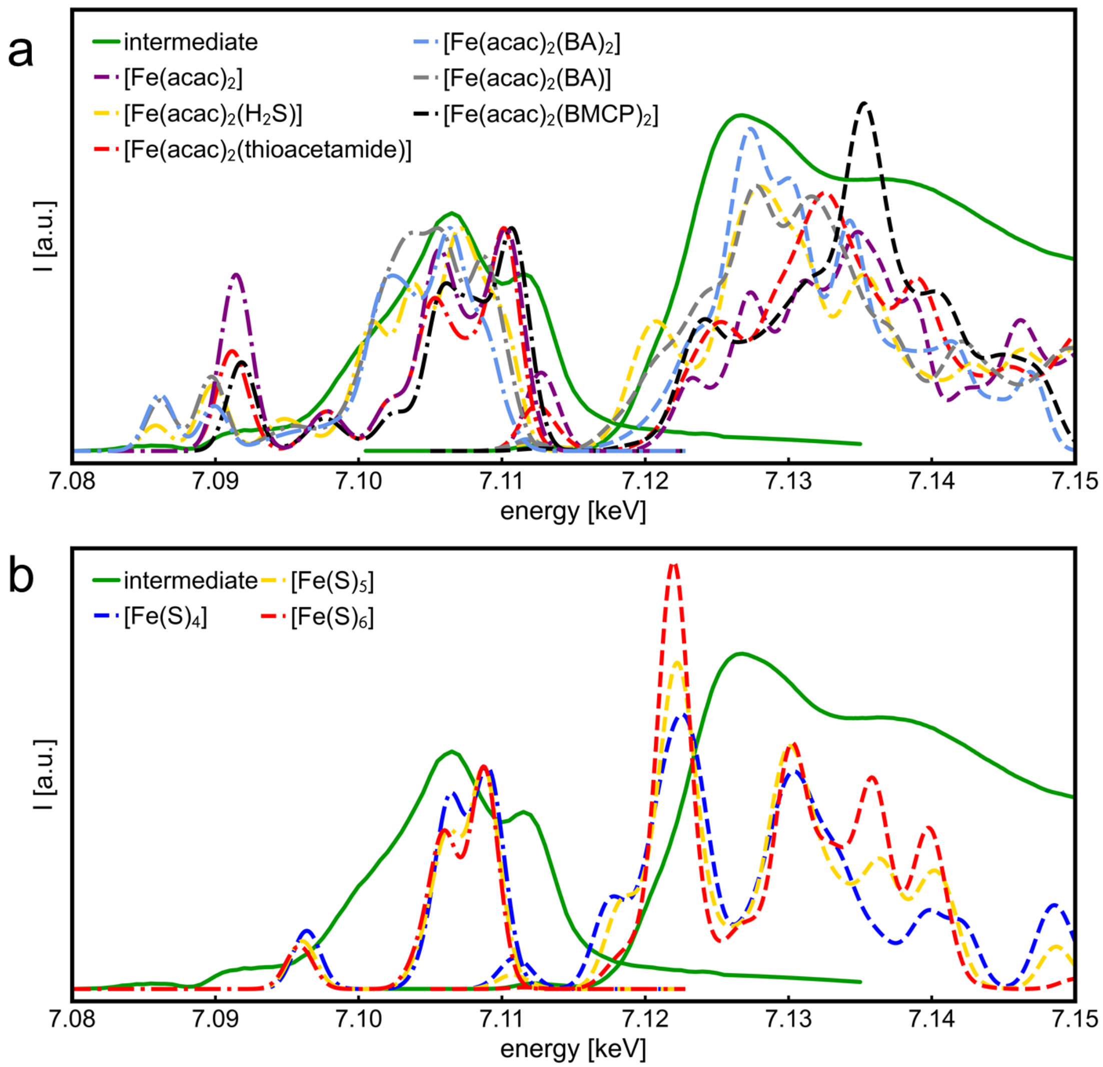

Figure S11. Identification of the first intermediate of the reaction, i.e., second recovered compound MCR-ALS analysis. Comparison between the experimental vtc-XES and HERFD-XANES data of the second recovered compound and theoretical spectra from DFT calculation of possible intermediates: **(a)** $Fe(acac)_2$ structures with different geometries and ligands, including H_2S, thioacetamide, BA, benzyl mercaptan (BMCP), or **(b)** tetrahedral FeS ($Fe(S)_4$), pyramidal FeS ($Fe(S)_5$), and octahedral FeS ($Fe(S)_6$) molecular cutouts, evidencing that the coordination of the intermediate compound does not correspond to sulfur-coordination. To align with the experimental data, the theoretical XANES spectra were shifted by 25.0 eV, and the vtc-XES spectra were shifted by 22.8 eV.

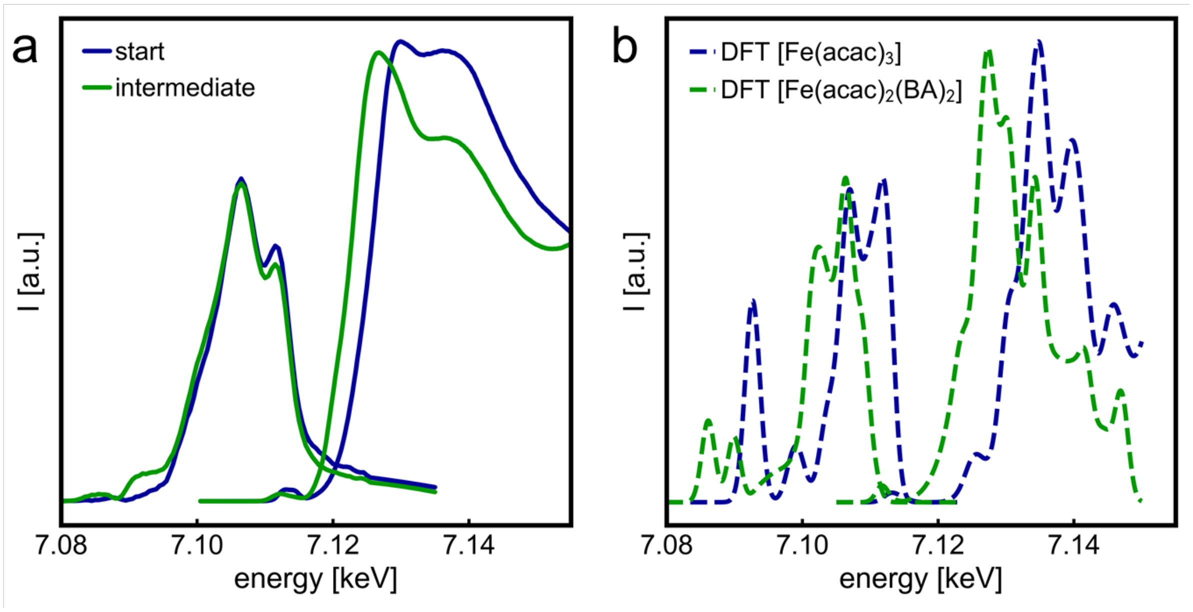

Figure S12. Comparison between the two molecular structures: $Fe(acac)_3$ and $[Fe(acac)_2(BA)_2]$, which are the first and second recovered components from MCR-ALS analysis. (a) Recovered HERFD-XANES spectra by MCR-ALS and vtc-XES spectra at the beginning of the reaction and at ~40 min reaction time (exclusively dominated by the second component according to MCR-ALS), **(b)** Theoretical XAS and vtc-XES from DFT calculations of a molecular unit of $Fe(acac)_3$ and $[Fe(acac)_2(BA)_2]$ complex. To align with the experimental data, the theoretical XANES spectra were shifted by 25.0 eV, while the vtc-XES spectra were shifted by 22.8 eV.

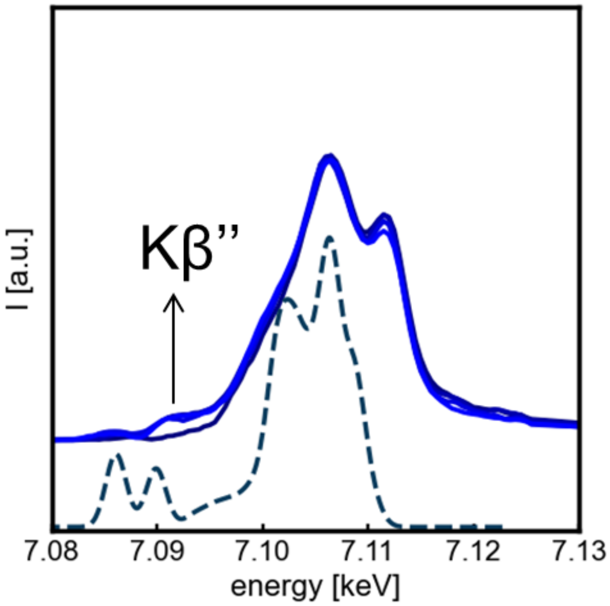

Figure S13. The emergence of the Kβ'' feature upon formation of the $[Fe(acac)_2(BA)_2]$ complex. Overlap of the first four scans of the *in situ* vtc-XES dataset during the synthesis of Fe_3S_4 at 180 °C (at 1 °C/min), compared with the DFT calculations of $[Fe(acac)_2(BA)_2]$ complex. The calculated vtc-XES spectrum was shifted by 22.8 eV to match to the experimental data.

Supplementary Notes 3: *In-situ* vtc-XES during the synthesis of Fe_3O_4 nanoparticles as a reference system

In **Figure 4g,h**, we demonstrate that vtc-XES captures the changes from the O- to S-coordination during the reaction evolution from $Fe(acac)_3$ to Fe_3S_4. However, the nature of the chemical bonding in the compounds differs significantly, i.e., a molecular complex *versus* a crystalline structure. To reveal the ligand identification in a similar chemical structure, we perform *in situ* vtc-XES measurements during the formation of Fe_3O_4 nanoparticles at 180 °C, using $Fe(acac)_3$ in BA, but without TAA as sulfur source (**Figure S14**).[12] As shown in **Figure S14a**, the two-peak $K\beta_{2,5}$ line at the beginning of the reaction, characteristic of $Fe(acac)_3$, converges into a single feature upon formation of Fe_3O_4 nanoparticles, similar to the behavior observed during the Fe_3S_4 formation. **Figure S14b** compares the Fe_3O_4 and Fe_3S_4 spectra, showing that the $K\beta_{2,5}$ line is much broader for Fe_3O_4 than for the Fe_3S_4. In parallel, the energy position of Kβ'' line also changes significantly, from 7099.5 eV in Fe_3S_4 to 7093.5 eV in Fe_3O_4, reflecting the higher covalency of S(3*s*) ligands compared to O(2*s*) ligands.[13-15] Additionally, FEFF simulations of Fe_3O_4 and Fe_3S_4 in **Figure S14c** produce the experimental differences. Therefore, we demonstrate that *in situ* vtc-XES enables the unambiguous identification of the ligand's nature, whether oxygen or sulfur, in solution and at elevated temperatures.

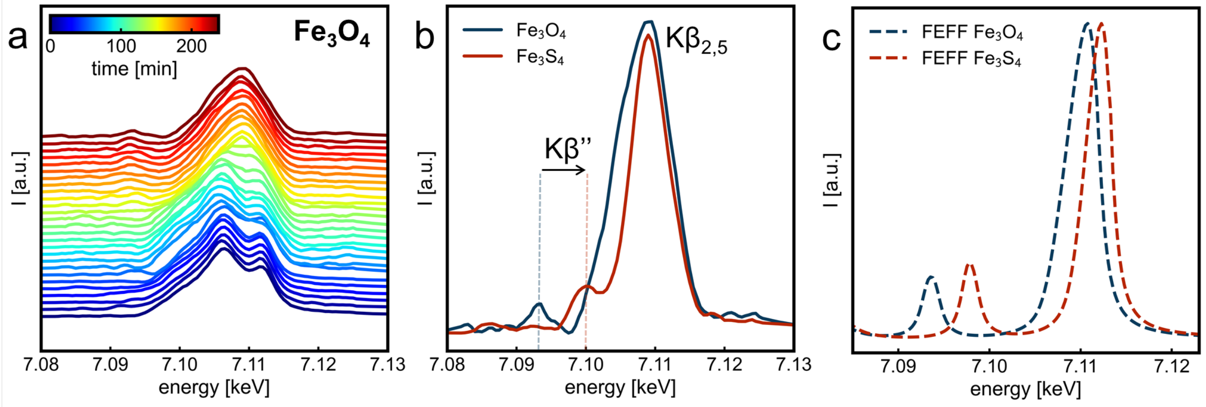

Figure S14. Study on the formation of Fe_3O_4 nanoparticles as a reference system. (a) *In situ* vtc-XES dataset collected during the synthesis of Fe_3O_4 at 180 °C and heating rate of 1 °C/min. The reaction was carried out using $Fe(acac)_3$ in benzyl alcohol without the addition of thioacetamide. **(b)** Comparison of the vtc-XES spectra of the final products Fe_3S_4 and Fe_3O_4, showing the shift in the Kβ'' due to the change in the ligand from O(2*s*) to S(3*s*). **(c)** Theoretical vtc-XES spectra of Fe_3S_4 and Fe_3O_4 from FEFF calculations. The absolute energies of the calculated spectra were shifted to align with experiment by using the parameters in Table S6.

Supplementary Notes 4: Pre-edge analysis

An extensive analysis of the pre-edge features in 3d transition metals has been provided in several reports.[16-18] In our study, we adopt the methodology proposed by Westre et al. for Fe compounds, which incorporates multiplet theory and many-electron effects.[19] In the K-edge XANES spectrum of transition metals such as Fe, the pre-edge originates from quadrupole-allowed 1*s* to 3*d* transitions. These transitions probe the unoccupied 3*d* orbitals that are split into different energy levels due to the surrounding ligand field. For instance, in an octahedral ligand field, the 3*d* orbitals split into lower-energy t_{2g} and higher-energy e_g levels, while in a tetrahedral field, the 3*d* orbitals split into lower-energy e and higher-energy t_2 levels (**Figure 5b**). The core excitation leads to a d^{n+1} configuration, and the resulting many-electron final states can be determined using ligand field theory. For each combination of symmetry, ligand-field strength, and electronic configuration, one can use the Tanabe-Sugano diagram, or Orgel diagram for high-spin cases, to stablish the possible final-state terms after the core excitation, each of which may contribute to the pre-edge feature.[16, 20] The possible excited states for Fe^{2+} and Fe^{3+} in octahedral and tetrahedral symmetry are shown in **Figure S15**.

The first component in our reaction corresponds to $Fe(acac)_3$, an octahedral high-spin Fe^{3+} complex. As depicted in **Figure S15a**, the 1*s*→3*d* electron excitation in Fe^{3+} (d^5 ground state, $^6A_{1g}$) results in two possible excited configurations: $\left|t_{2g}^4 e_g^2\right\rangle$ and $\left|t_{2g}^3 e_g^3\right\rangle$. These configurations generate the spin-allowed final states $^5T_{2g}$ and ^5E_g, respectively, which contribute to the two pre-edge features seen in **Figure 5c**. Since these two final states represent configurations in which the excited electron occupies either a t_{2g} or e_g orbital and the 3*d* spin-orbit interaction does not impact the energies, the energy difference between the two pre-edge peaks provides a direct experimental measure of the octahedral crystal-field splitting parameter (Δ_{oct} or 10Dq).

In the case of the molecular intermediate $[Fe(acac)_2(BA)_2]$ complex, we assume a mostly centrosymmetric environment for Fe^{2+} in the octahedral sites (O_h). The electron excitation from 1*s* to 3*d* orbitals in the high-spin Fe^{2+} (d^6 ground state, $^5T_{2g}$) allows different excited configurations, namely $\left|t_{2g}^5 e_g^2\right\rangle$ and $\left|t_{2g}^4 e_g^3\right\rangle$, as shown in **Figure S15b**. While the $\left|t_{2g}^5 e_g^2\right\rangle$ configuration generates the $^4T_{1g}$ state, the $\left|t_{2g}^4 e_g^3\right\rangle$ configuration results in the $^4T_{2g}$ and $^4T_{1g}$ states. These correspond to the only spin-allowed transitions that contribute to the pre-edge, however, other spin-forbidden multiplet states can arise due to spin coupling.[19] Therefore, three pre-edge features are expected, as seen in **Figure 5e**. The energy difference between the $^4T_{1g}$ at the lowest energy and the $^4T_{2g}$ gives only an approximation of the Δ_{oct} in the molecular complex,[16] as the 3*d* spin-orbit coupling effect is more impactful than in Fe^{3+}, and, thus, the $^4T_{1g}$ final states have contributions of both e_g and t_{2g} orbitals.[21]

The pre-edge of FeS mackinawite is the most intense among the four components in the reaction as it arises from the Fe^{2+} ions fully in T_d sites, lacking inversion center and allowing both quadrupole and dipole transitions. The mixing of $4p$ and $3d$ orbitals is allowed by symmetry rules, where the $4p$ orbitals can interact only with the t_2 orbitals. **Figure S15c** displays the possible states from the core excitation for Fe^{2+} in T_d sites (d^6 ground state, ^5E). The electron excitation to the e orbital results in the $|e^4t_2^3\rangle$ excited configuration, which generates the final state $^4A_2$, whereas the excitation to the t_2 orbital generates the $|e^3t_2^4\rangle$ excited configuration state that gives rise to the $^4T_2$ and $^4T_1$ states. Another $^4T_1$ state is generated by the excitation of two electrons, promoting the $|e^2t_2^5\rangle$ excited configuration. Nevertheless, this second $^4T_1$ state also contains the allowed $|e^3t_2^4\rangle$ component provided by the mixing with the other $^4T_1$ state caused by symmetry. In this case, the $^4T_2$ and both $^4T_1$ final states have a hole in the *e* orbital for the excited states (i.e., $|e^3t_2^4\rangle$), allowing the $4p$-$3d$ hybridization according to the group theory, which originates the electric dipole-allowed contributions.[19] In our experimental data, the first peak at lower energy corresponds to overlapping transitions to multiple unresolved final states, including $^4A_2$, $^4T_2$, and $^4T_1$ states, which cannot be resolved due to the intrinsic experimental resolution of ~1.4 eV, but the intensity is mostly dominated by the $^4T_2$ state, while the second, higher-energy peak is primarily assigned to the $^4T_1$ state (**Figure 5f**).

In Fe_3S_4 greigite, Fe ions exist in three different oxidation states and symmetries, one third corresponds to Fe^{3+} in T_d sites, one-third is Fe^{2+} in O_h sites, and one-third is Fe^{3+} in O_h sites. Therefore, the K pre-edge should contain multiple origins of each site. The Fe^{2+} in O_h symmetry would generate three final states, similar to the $[Fe(acac)_2(BA)_2]$ complex, while the Fe^{3+} in O_h could generate two final states similarly to $Fe(acac)_3$ (see **Figure S15a,b**). As evidenced in **Figure S15d**, the excited states for Fe^{3+} in T_d (d^5 ground state, $^6A_1$) allows the $|e^3t_2^3\rangle$ and $|e^2t_2^4\rangle$ excited configurations, which yields the $^5T_2$ and ^5E many electron-states, respectively.[16] The intensity related to the $^5T_2$ increases as it is dipole-allowed by the interaction of $4p$ orbitals with the t_2 orbitals. These two states, however, cannot be resolved in the K pre-edge and a single peak is expected for Fe^{3+} in T_d symmetry.[19, 21] Consequently, one would expect six distinct peaks to appear for Fe_3S_4; however, these likely overlap and remain unresolved due to the limited energy resolution. Instead, the final states with the highest intensity should dominate the pre-edge features. Thus, the experimental data for Fe_3S_4 is resolved into three components, which are probably from the Fe^{3+} in T_d symmetry (final state $^5T_2$), Fe^{2+} in O_h sites ($^4T_{1g}$ final state), and Fe^{3+} in O_h sites (^5E_g final state). However, other contributions cannot be ruled out due to overlapping, such as a small contribution of the second $^4T_{1g}$ final state from Fe^{2+} in O_h symmetry.

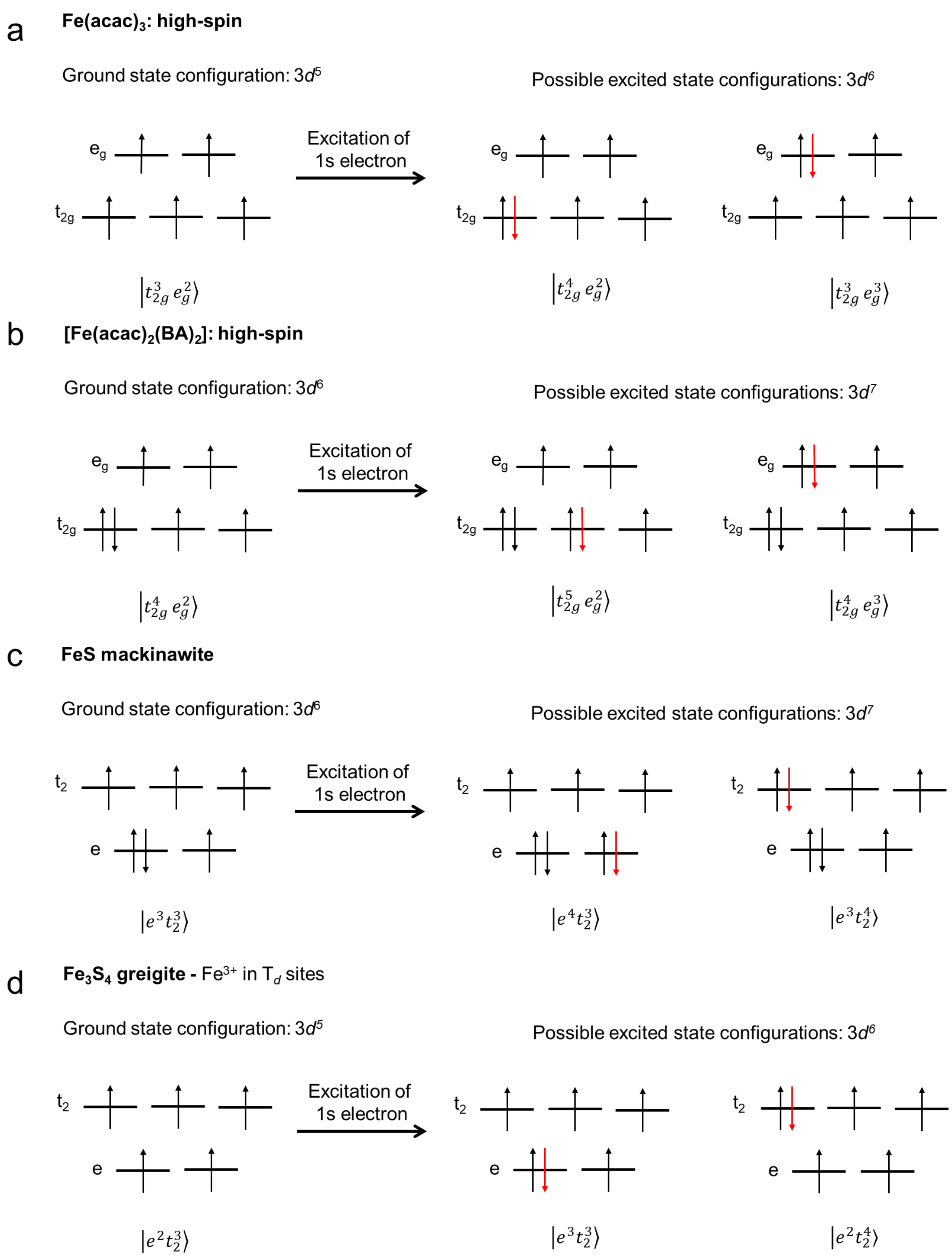

Figure S15. Schematic illustration of the 3*d* orbitals in the ground state and possible final states after the core 1*s* electron excitation for the Fe ions in different oxidation states and symmetry: **(a)** Fe^{3+} in a high-spin O_h symmetry such as in the $Fe(acac)_3$, **(b)** Fe^{2+} in O_h symmetry and high-spin, like in the $[Fe(acac)_2(BA)_2]$ intermediate complex, **(c)** Fe^{2+} T_d symmetry in FeS mackinawite, and **(d)** Fe^{3+} in T_d symmetry in Fe_3S_4 greigite, while the other sites for Fe_3S_4 can be represented by (a,b) configurations.

Table S5. Pre-edge fit for the four recovered components by MCR-ALS analysis.

Components	Oxidation state and symmetry	Centroid position (eV)	Peaks (eV)	Component splitting (eV)[a]
$Fe(acac)_3$	Fe^{3+} (O_h)	7113.38	7112.78<br>7114.34	1.56
$[Fe(acac)_2(BA)_2]$	Fe^{2+} (O_h)	7112.54	7111.96<br>7113.20<br>7114.46	2.5
FeS	Fe^{2+} (T_d)	7112.45	7112.11<br>7113.50	1.39
Fe_3S_4	1/3 Fe^{3+} (T_d)<br>2/3 Fe^{3+}/Fe^{2+} (O_h)	7113.26	7111.91<br>7113.01<br>7114.58	2.67

[a] The energy difference corresponds to the energy difference between the lowest energy and highest energy peaks.

We validate the fitting of components recovered from the MCR-ALS method by comparing them with fits obtained from the resonant inelastic X-ray scattering (RIXS) maps. We collected 1*s*2*p* RIXS maps for both the starting point of the reaction (before heating) and the final product, Fe_3S_4, after 60 min at 180 °C, as shown in **Figure S16a,b**. By performing the constant emission energy (CEE) cut, indicated by the white dashed line in the RIXS maps, we obtain the corresponding pre-edge spectra shown in **Figure S16c,d**. The pre-edge spectrum of the starting point of the reaction (i.e., $Fe(acac)_3$) shows the centroid at 7113.24 eV with two features at 7112.81 eV ($^4T_{2g}$) and 7114.41 eV (^4E_g), giving a ΔE of 1.6 eV. For the final product (Fe_3S_4), the pre-edge fit reveals three components at 7111.70 eV (Fe^{2+} in O^h), 7112.66 eV (Fe^{3+} in T_d) and 7114.22 eV (Fe^{3+} in O_h), with the centroid seen at 7112.65 eV. These results are similar to the fitted data retrieved from the MCR-ALS method, demonstrating the reliability of the pre-edge fitting of the spectra obtained through MCR-ALS analysis.

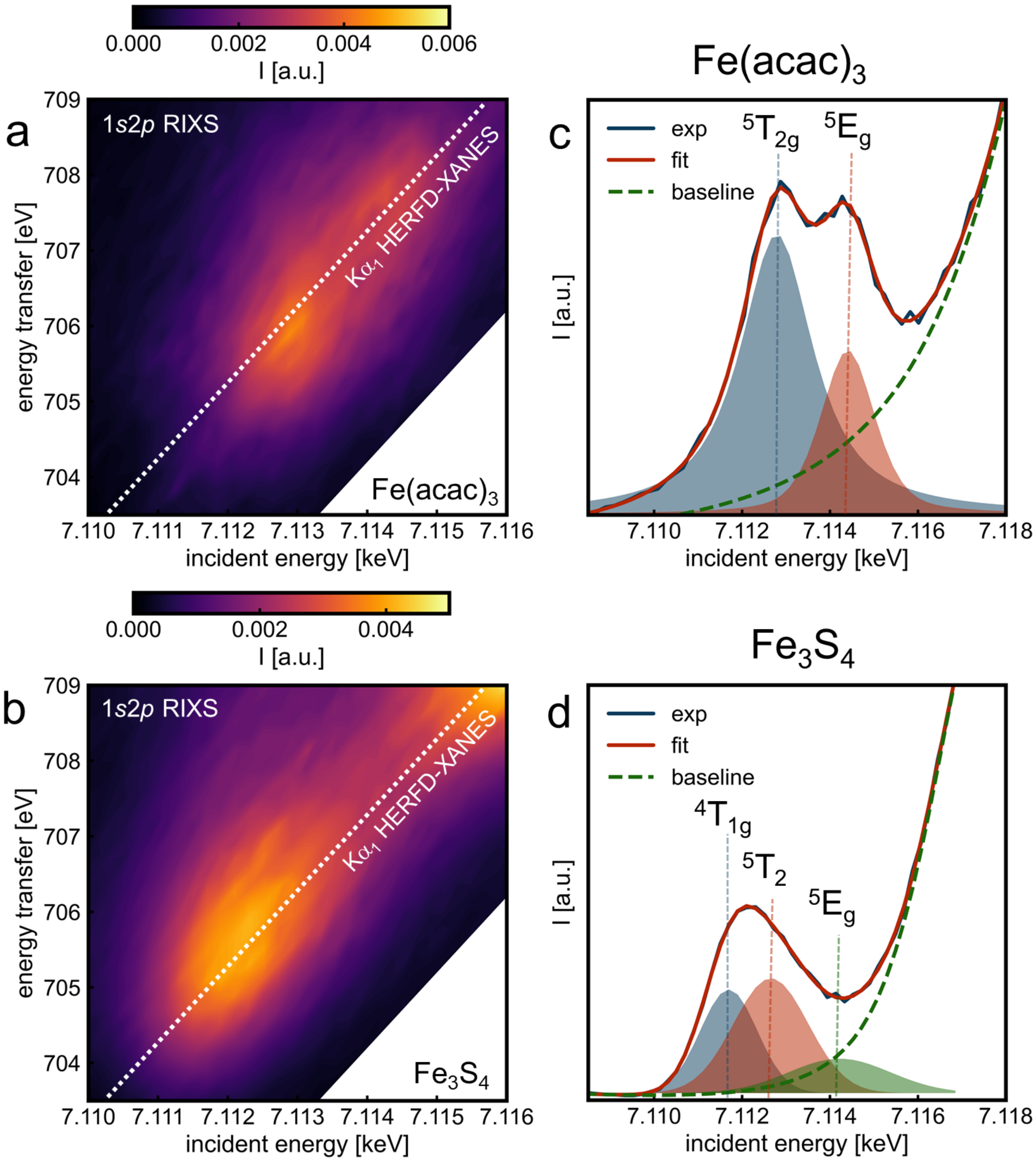

Figure S16. Analysis of the pre-edge features from RIXS maps. (a,b) Experimental 1*s*2*p* RIXS maps of **(a)** precursor solution of Fe(acac)$_3$ and TAA in BA, and **(b)** final reaction product, Fe_3S_4, in BA solution. The white dashed lines correspond to the CEE cut. **(c,d)** Corresponding K-edge spectra obtained from the CEE cuts of RIXS maps along with the fits of the pre-edge contributions into the different final states for **(c)** precursor in solution (Fe(acac)$_3$), and **(d)** final product Fe_3S_4 in solution.

Table S6. Input settings for the FEFF calculations of XANES and vtc-XES spectra.

XANES	vtc-XES
EDGE K<br>S02 1.0<br>CONTROL 1 1 1 1 1 1<br>PRINT 1 0 0 0 0 3<br>COREHOLE FSR<br>MULTIPOLE 2 0<br>SETEDGE<br>EGRID<br>e_grid -15 120.0 0.5<br>EXCHANGE 0 1 0 -1<br>LDOS -20 30 0.5<br>XANES 10 0.05 0.05<br>FMS 9 0	EDGE K<br>S02 1.0<br>CONTROL 1 1 1 1 1 1<br>PRINT 1 0 0 0 0 3<br>SCF 6 1 100 0.2 1<br>COREHOLE RPA<br>MULTIPOLE 2 0<br>XES 7120 7085 0.25<br>EXCHANGE 5 0 0.2 -1<br>SETEDGE<br>LDOS -40 0 0.2<br>REAL

Table S7. ORCA input files for structural optimization and calculations of XANES and vtc-XES data.

XANES	vtc-XES
!RKS B3LYP TightSCF ZORA-def2-TZVP D3BJ RIJCOSX ZORA<br>!Normalprint MOREAD<br>%maxcore 10000<br>%moinp "/orca_XES.gbw"<br>%tddft<br>orbwin[0]= 0,0,-1,-1 doquad true<br>nroots 300<br>maxdim 50<br>end<br>…	!RKS B3LYP TightOpt TightSCF ZORA-def2-TZVP ZORA<br>!FREQ Largeprint Printbasis<br>%PAL NPROCS 16 END<br>%maxcore 20000<br>%xes<br>CoreOrb 0<br>OrbOp 0<br>end<br>…

Supplementary references

(1) Lyubutin, I. S.; Starchikov, S. S.; Lin, C.-R.; Lu, S.-Z.; Shaikh, M. O.; Funtov, K. O.; Dmitrieva, T. V.; Ovchinnikov, S. G.; Edelman, I. S.; Ivantsov, R. Magnetic, structural, and electronic properties of iron sulfide Fe3S4 nanoparticles synthesized by the polyol mediated process. *Journal of Nanoparticle Research* **2013**, *15* (1), 1397. DOI: 10.1007/s11051-012-1397-0.

(2) Paolella, A.; George, C.; Povia, M.; Zhang, Y.; Krahne, R.; Gich, M.; Genovese, A.; Falqui, A.; Longobardi, M.; Guardia, P.; et al. Charge Transport and Electrochemical Properties of Colloidal Greigite (Fe3S4) Nanoplatelets. *Chemistry of Materials* **2011**, *23* (16), 3762-3768. DOI: 10.1021/cm201531h.

(3) Garnero, C.; Lepesant, M.; Garcia-Marcelot, C.; Shin, Y.; Meny, C.; Farger, P.; Warot-Fonrose, B.; Arenal, R.; Viau, G.; Soulantica, K.; et al. Chemical Ordering in Bimetallic FeCo Nanoparticles: From a Direct Chemical Synthesis to Application As Efficient High-Frequency Magnetic Material. *Nano Letters* **2019**, *19* (2), 1379-1386. DOI: 10.1021/acs.nanolett.8b05083.

(4) Krycka, K. L.; Borchers, J. A.; Booth, R. A.; Ijiri, Y.; Hasz, K.; Rhyne, J. J.; Majetich, S. A. Origin of Surface Canting within Fe_3O_4 Nanoparticles. *Physical Review Letters* **2014**, *113* (14), 147203. DOI: 10.1103/PhysRevLett.113.147203.

(5) Li, G.; Zhang, B.; Yu, F.; Novakova, A. A.; Krivenkov, M. S.; Kiseleva, T. Y.; Chang, L.; Rao, J.; Polyakov, A. O.; Blake, G. R.; et al. High-Purity Fe3S4 Greigite Microcrystals for Magnetic and Electrochemical Performance. *Chemistry of Materials* **2014**, *26* (20), 5821-5829. DOI: 10.1021/cm501493m.

(6) Beal, J. H. L.; Prabakar, S.; Gaston, N.; Teh, G. B.; Etchegoin, P. G.; Williams, G.; Tilley, R. D. Synthesis and Comparison of the Magnetic Properties of Iron Sulfide Spinel and Iron Oxide Spinel Nanocrystals. *Chemistry of Materials* **2011**, *23* (10), 2514-2517. DOI: 10.1021/cm2002868.

(7) Cao, F.; Hu, W.; Zhou, L.; Shi, W.; Song, S.; Lei, Y.; Wang, S.; Zhang, H. 3D Fe3S4 flower-like microspheres: high-yield synthesis via a biomolecule-assisted solution approach, their electrical, magnetic and electrochemical hydrogen storage properties. *Dalton Transactions* **2009**, (42), 9246-9252, 10.1039/B912569H. DOI: 10.1039/B912569H.

(8) Roberts, A. P.; Chang, L.; Rowan, C. J.; Horng, C.-S.; Florindo, F. Magnetic properties of sedimentary greigite (Fe3S4): An update. *Reviews of Geophysics* **2011**, *49* (1). DOI: https://doi.org/10.1029/2010RG000336 (acccessed 2025/05/15).

(9) Grote, L.; Zito, C. A.; Frank, K.; Dippel, A.-C.; Reisbeck, P.; Pitala, K.; Kvashnina, K. O.; Bauters, S.; Detlefs, B.; Ivashko, O.; et al. X-ray studies bridge the molecular and macro length scales during the emergence of CoO assemblies. *Nature Communications* **2021**, *12* (1), 4429. DOI: 10.1038/s41467-021-24557-z.

(10) Klemeyer, L.; Gröne, T. L. R.; Zito, C. d. A.; Vasylieva, O.; Gumus Akcaalan, M.; Harouna-Mayer, S. Y.; Caddeo, F.; Steenbock, T.; Hussak, S.-A.; Kesavan, J. K.; et al. Utilizing High X-ray Energy Photon-In Photon-Out Spectroscopies and X-ray Scattering to Experimentally Assess the Emergence of Electronic and Atomic Structure of ZnS Nanorods. *Journal of the American Chemical Society* **2024**, *146* (49), 33475-33484. DOI: 10.1021/jacs.4c10257.

(11) Staniuk, M.; Hirsch, O.; Kränzlin, N.; Böhlen, R.; van Beek, W.; Abdala, P. M.; Koziej, D. Puzzling Mechanism behind a Simple Synthesis of Cobalt and Cobalt Oxide Nanoparticles: In Situ Synchrotron X-ray Absorption and Diffraction Studies. *Chemistry of Materials* **2014**, *26* (6), 2086-2094. DOI: 10.1021/cm500090r.

(12) Pinna, N.; Grancharov, S.; Beato, P.; Bonville, P.; Antonietti, M.; Niederberger, M. Magnetite Nanocrystals: Nonaqueous Synthesis, Characterization, and Solubility. *Chemistry of Materials* **2005**, *17* (11), 3044-3049. DOI: 10.1021/cm050060+.
(13) Vegelius, J. R.; Kvashnina, K. O.; Klintenberg, M.; Soroka, I. L.; Butorin, S. M. Cu Kβ2,5 X-ray emission spectroscopy as a tool for characterization of monovalent copper compounds. *Journal of Analytical Atomic Spectrometry* **2012**, *27* (11), 1882-1888, 10.1039/C2JA30095H. DOI: 10.1039/C2JA30095H.
(14) Lee, N.; Petrenko, T.; Bergmann, U.; Neese, F.; DeBeer, S. Probing Valence Orbital Composition with Iron Kβ X-ray Emission Spectroscopy. *Journal of the American Chemical Society* **2010**, *132* (28), 9715-9727. DOI: 10.1021/ja101281e.
(15) Mortensen, D. R.; Seidler, G. T.; Kas, J. J.; Govind, N.; Schwartz, C. P.; Pemmaraju, S.; Prendergast, D. G. Benchmark results and theoretical treatments for valence-to-core x-ray emission spectroscopy in transition metal compounds. *Physical Review B* **2017**, *96* (12), 125136. DOI: 10.1103/PhysRevB.96.125136.
(16) Calas, G.; Petiau, J. Coordination of iron in oxide glasses through high-resolution K-edge spectra: Information from the pre-edge. *Solid State Communications* **1983**, *48* (7), 625-629. DOI: https://doi.org/10.1016/0038-1098(83)90530-6.
(17) Vercamer, V.; Hunault, M. O. J. Y.; Lelong, G.; Haverkort, M. W.; Calas, G.; Arai, Y.; Hijiya, H.; Paulatto, L.; Brouder, C.; Arrio, M.-A.; et al. Calculation of optical and K pre-edge absorption spectra for ferrous iron of distorted sites in oxide crystals. *Physical Review B* **2016**, *94* (24), 245115. DOI: 10.1103/PhysRevB.94.245115.
(18) Arrio, M. A.; Rossano, S.; Ch, B.; Galoisy, L.; Calas, G. Calculation of multipole transitions at the Fe K pre-edge through p-d hybridization in the Ligand Field Multiplet model. *Europhysics Letters* **2000**, *51* (4), 454. DOI: 10.1209/epl/i2000-00515-8.
(19) Westre, T. E.; Kennepohl, P.; DeWitt, J. G.; Hedman, B.; Hodgson, K. O.; Solomon, E. I. A Multiplet Analysis of Fe K-Edge 1s → 3d Pre-Edge Features of Iron Complexes. *Journal of the American Chemical Society* **1997**, *119* (27), 6297-6314. DOI: 10.1021/ja964352a.
(20) Zimmermann, P.; Hunault, M. O. J. Y.; de Groot, F. M. F. 1s2p RIXS Calculations for 3d Transition Metal Ions in Octahedral Symmetry. *Journal of Spectroscopy* **2018**, *2018* (1), 3618463. DOI: https://doi.org/10.1155/2018/3618463 (acccessed 2025/07/23).
(21) Guo, M.; Sørensen, L. K.; Delcey, M. G.; Pinjari, R. V.; Lundberg, M. Simulations of iron K pre-edge X-ray absorption spectra using the restricted active space method. *Physical Chemistry Chemical Physics* **2016**, *18* (4), 3250-3259, 10.1039/C5CP07487H. DOI: 10.1039/C5CP07487H.
